# Comparative Stabilization of TSC Composite Soil Using Cement, Lime, Solid-Waste-Based Binders, and MICP: Mechanical Performance, Microstructure, and Carbon Emissions

**DOI:** 10.3390/ma19214636

**Published:** 2026-10-31

**Authors:** Haowei Ding, Qiwei Zhan, Changhao Fu, Wenqian Gan

**Affiliations:** 1 School of Civil Engineering and Architecture, Jiangsu University of Science and Technology, Zhenjiang 212000, China; 241710901101@stu.just.edu.cn (H.D.); 261210901101@stu.just.edu.cn (W.G.); 2 Zhenjiang Key Laboratory of Infrastructure Renewal and Resilience Enhancement, Zhenjiang 212000, China; 3 School of Transportation, Southeast University, Nanjing 211189, China

**Keywords:** titanium gypsum, soil stabilization, microstructural characterization, freeze–thaw durability, life cycle assessment, biomineralization

## Abstract

To promote the resource utilization of industrial by-product titanium gypsum while improving its engineering performance, a TSC composite soil containing titanium gypsum and clay at a mass ratio of 60:40 was investigated. Four stabilization methods, namely cement, solid-waste-based cementitious binder, lime, and microbially induced calcium carbonate precipitation (MICP), were systematically compared. Mechanical and durability performance was evaluated through unconfined compressive strength, water-stability, and freeze–thaw tests at different curing ages, while the stabilization mechanisms were characterized using X-ray diffraction (XRD), Fourier-transform infrared spectroscopy (FTIR), scanning electron microscopy coupled with energy-dispersive spectroscopy (SEM-EDS), and nitrogen adsorption–desorption analysis (BET). Carbon emissions were further evaluated using a life-cycle-based emission-factor approach. The comparative framework integrating engineering performance, microstructural mechanisms, and environmental impacts constitutes the main contribution of this study. At 28 days, cement-stabilized soil exhibited the highest UCS of approximately 2.6 MPa, followed by the solid-waste-based binder, lime, and MICP systems. MICP produced CaCO_3_ precipitates that filled pores and formed interparticle bridging structures, thereby improving water stability and structural integrity despite its relatively lower short-term strength. The estimated net carbon emission of the MICP system was 4.3 kg CO_2_/t of stabilized soil, approximately 86% lower than that of cement stabilization. These results demonstrate a clear trade-off between mechanical performance and environmental impact among the four stabilization approaches and indicate that MICP is a promising low-carbon option for titanium-gypsum-based composite soils, although further field-scale and long-term validation is required.

## 1. Introduction

With the continuous expansion of infrastructure construction, the demand for filling materials in subgrade engineering has increased significantly [1]. By 2025, the annual consumption of earthwork materials for highway construction in China was projected to exceed 2 billion tons. The intensive exploitation of natural clay resources has exacerbated soil erosion and increased geological environmental risks [2]. Meanwhile, the large-scale generation and long-term accumulation of industrial solid waste have become major environmental challenges. Therefore, promoting the coordinated development of industrial by-product utilization and soil improvement technologies has become an important approach to achieving the goals of the 2030 Agenda for Sustainable Development [3], as well as resource recycling and green infrastructure construction [4]. TSC composite soil, formed by blending industrial by-product titanium gypsum with natural clay, represents a new material system with potential for both waste utilization and performance regulation. However, untreated TSC composite soil still exhibits several limitations, including low dry density, poor volumetric stability, and limited strength, making it difficult to satisfy the bearing capacity and durability requirements of subgrade engineering [5]. Although traditional stabilizing materials such as cement and lime can significantly improve soil strength, their production processes are associated with high carbon emissions and substantial energy consumption [6], which contradicts the goals of green and low-carbon development. Therefore, developing environmentally sustainable stabilization technologies for TSC composite soil that achieve synergistic optimization of engineering performance and environmental benefits is of significant theoretical and practical importance.

In the field of composite soil material preparation and engineering applications, G. Zenghuan et al. first utilized the industrial by-product phosphogypsum (PG) as the primary raw material, with slag (SG) and Portland cement (PC) as auxiliary binders. The prepared specimens achieved a 60-day compressive strength of 28.8 MPa, a softening coefficient of 0.9, and an expansion rate of approximately −0.2% [7]. J. James et al. investigated the use of industrial wastes as auxiliary additives in cement- and lime-stabilized soils and confirmed that lime stabilization is the preferred method for plastic clays, although it is ineffective for sulfate-rich clays and performs poorly under extreme environmental conditions [8]. Y. Zhou et al. employed cement–slag-based binders to stabilize soils with different properties and revealed that strength development mainly results from the synergistic effects of particle cementation and pore filling by expansive hydration products, with the 28-day strength reaching 6.4 MPa under optimal dosage [9]. These studies demonstrate the potential of conventional and solid-waste-based binders for improving soil engineering performance. However, the effectiveness of different stabilization systems depends on the characteristics of the raw materials, binder composition, and curing conditions. Further comparative investigation is therefore needed to evaluate their mechanical performance, durability, and environmental impacts within the same composite-soil system.

In recent years, microbially induced calcium carbonate precipitation (MICP) [10] has emerged as a promising environmentally friendly soil stabilization technique. Building on this approach, A. M. Sharaky et al. optimized field application techniques for microbial cementation, significantly improving the adsorption efficiency and cementation performance of *Sporosarcina pasteurii* in sand [11]. Nevertheless, the application of MICP in composite soils with complex compositions is still at an exploratory stage. In addition to mechanical performance and durability, the evaluation of carbon emissions and environmental benefits has become an important research dimension, with life cycle assessment (LCA) widely adopted as a key method for assessing the environmental performance of stabilization technologies [12]. Furthermore, B. Shu et al. analyzed the carbon emission characteristics of solid-waste-based stabilization materials and demonstrated their low-carbon potential and engineering applicability through multi-indicator coupled analysis [13]. These studies highlight the importance of evaluating soil stabilization technologies from both engineering and environmental perspectives. Nevertheless, the applicability of existing findings to titanium-gypsum-based composite soil remains uncertain because of differences in material composition and stabilization mechanisms.

Although considerable progress has been made in composite soil material preparation and soil stabilization technologies, several key challenges remain in practical applications and technological development. The inherent engineering limitations of TSC composite soil, such as high expansibility and low density [14], have not yet been effectively addressed through existing stabilization approaches. Existing technologies struggle to simultaneously satisfy engineering requirements for both mechanical strength and long-term stability. Moreover, the high carbon emissions associated with traditional stabilization technologies remain a critical concern [15]. In addition, the long-term durability of solid-waste-based stabilization technologies [16] and the applicability of MICP in composite soils with complex compositions still require extensive experimental validation [17]. At present, systematic comparative studies evaluating different stabilization techniques in terms of mechanical performance, microstructural mechanisms, carbon emissions, and environmental benefits remain limited, making it difficult to develop targeted optimization strategies for different engineering scenarios. Furthermore, the optimization of reaction mechanisms and operational conditions for MICP in TSC composite soil, as well as the economic feasibility of its engineering application, requires further in-depth investigation. These gaps indicate the need for a systematic comparison of different stabilization approaches using a common TSC composite-soil matrix, with particular attention to the relationships among stabilization mechanisms, engineering performance, durability, and carbon emissions.

To address these research gaps, this study systematically compares four stabilization approaches—cement, solid-waste-based cementitious materials, lime, and MICP—using TSC composite soil prepared by blending titanium gypsum and clay at a mass ratio of 60:40. Unlike previous studies focusing primarily on individual stabilizers, this study integrates mechanical-performance testing, water-stability assessment, freeze–thaw testing, multi-technique microstructural characterization, and carbon-emission assessment within a unified comparative framework. The effects of the four stabilization approaches on the mechanical properties and durability of TSC composite soil are investigated, while microstructural analyses are used to examine the relationships between the observed engineering performance and the formation and distribution of stabilization products. Meanwhile, LCA is employed to compare the estimated carbon emissions of the different stabilization systems under the adopted assessment assumptions. By integrating these analyses, this study aims to clarify the differences among conventional, solid-waste-based, and biologically mediated stabilization approaches, evaluate the trade-offs between engineering performance and environmental impact, and identify potential pathways for the sustainable stabilization of TSC composite soil. The findings are expected to provide a comparative basis for the engineering utilization of industrial by-product titanium gypsum and the development of sustainable soil stabilization technologies.

Based on the identified research gaps and the objectives of this study, four hypotheses are formulated: (H1) the four stabilization methods exhibit different mechanical-performance and durability responses because of their distinct stabilization mechanisms; (H2) cement and solid-waste-based binders provide greater short-term strength and freeze–thaw resistance than lime and MICP treatment under the tested conditions; (H3) differences in macroscopic engineering performance are associated with the formation and distribution of cementitious or carbonate products and their effects on particle bonding and pore filling; and (H4) solid-waste-based and MICP stabilization exhibit lower estimated carbon emissions than conventional cement and lime stabilization under the adopted assessment assumptions.

## 2. Materials and Methods

### 2.1. Raw Materials

#### 2.1.1. Test Soil

**Figure 1:**
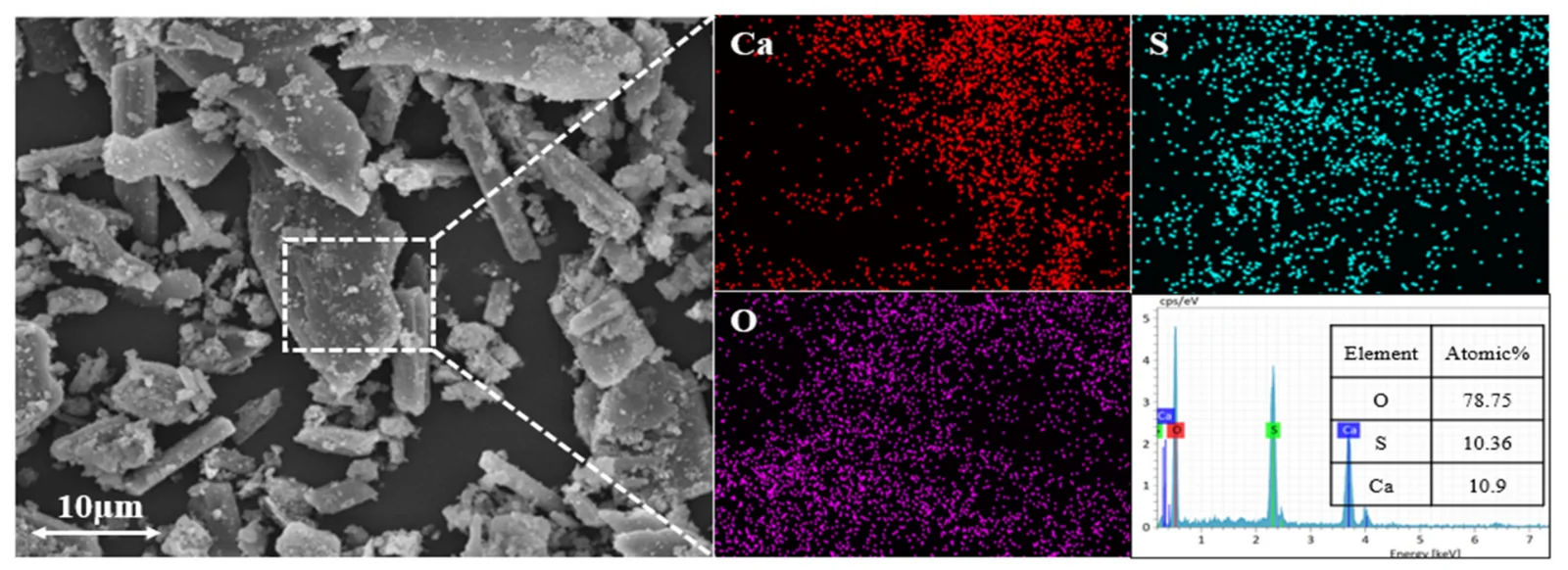
SEM and EDS images of titanium gypsum.

In this study, titanium gypsum (TG) and clay were selected as the primary raw materials. The titanium gypsum was obtained as an industrial by-product generated during the sulfuric acid process of titanium dioxide production at a plant located in Zhenjiang, Jiangsu Province, China, and served as the primary research material in this study. The clay was collected from soft soil deposits along river embankments in the Zhenjiang area. Both materials are readily available and representative of typical engineering application conditions. To determine the chemical composition of the two raw materials and ensure the reliability of subsequent modification experiments, X-ray fluorescence (XRF) analysis was conducted on titanium gypsum and clay [18]. The results are presented in Table 1. As shown in Table 1, titanium gypsum mainly consists of SO_3_, CaO, Fe_2_O_3_, and SiO_2_, which is consistent with the typical composition of industrial by-product titanium gypsum [18]. The clay is primarily composed of SiO_2_, Al_2_O_3_, and Fe_2_O_3_, with SiO_2_ accounting for the highest proportion (62.36%), which contributes to its inherent structural stability. To complement the XRF analysis, scanning electron microscopy (SEM) coupled with energy-dispersive spectroscopy (EDS) was employed [19], and the results are presented in Figure 1. The EDS results indicate that titanium gypsum is primarily composed of O, S, and Ca. These findings are consistent with the chemical composition identified by the XRF analysis, confirming the reliability of the compositional characterization of the raw materials.

After determining the source and chemical composition of the raw materials, systematic tests were conducted to evaluate the basic engineering properties of pure titanium gypsum. The results indicate that pure titanium gypsum exhibits significant limitations in key parameters, including pH value, liquid limit (LL), plastic limit (PL) [20], dry density, and free swelling ratio (FSR), which restrict its direct application in engineering practice [21]. The pH value of titanium gypsum was 9.46, while the liquid and plastic limits were 67.82% and 30.96%, respectively, indicating strong water absorption, swelling potential, and plasticity. Its dry density was 1128.79 kg·m^−3^, significantly lower than that of clay (1582.10 kg·m^−3^), suggesting a relatively loose structure and low compactness. In addition, the free swelling ratio reached 42%, which is significantly higher than that of conventional clay, indicating pronounced expansibility and poor volumetric stability. To address these engineering limitations, a composite modification strategy was adopted. Based on preliminary tests, the optimal mass ratio of titanium gypsum to natural clay was determined to be 60:40, and the composite soil prepared at this ratio was denoted as TSC. After blending, the particle-size distribution of the soil was optimized, resulting in a more stable structure and significant improvements in both plasticity characteristics and volumetric stability. The basic physical properties of the TSC composite soil are presented in Table 2. The liquid and plastic limits were 46.71% and 23.91%, respectively; the dry density increased to 1427.03 kg·m^−3^, while the free swelling ratio decreased to 29.5%. Compared with pure titanium gypsum, the plasticity index of the composite system decreased significantly, the structural compactness increased markedly, and the expansibility was effectively suppressed. These results indicate that clay blending effectively improves the engineering applicability of titanium gypsum and provides a stable matrix for subsequent stabilization modification tests.

#### 2.1.2. Selection of Curing Agent

To enhance the mechanical properties and long-term stability of TSC composite soil, four representative curing agents were selected: cement, lime, solid waste-based cementitious materials, and microbial curing agents. A systematic comparative experiment was conducted. The cement used in this study was P·O 42.5 ordinary Portland cement produced by Cangrun Building Materials Co., Ltd., Jiangsu, China, which complies with the Chinese standard General Portland Cement (GB 175-2023) [22]. The cement has an initial setting time of ≥45 min and a final setting time of ≤6.5 h, while the compressive strengths at 3 and 28 days are ≥17.0 MPa and ≥42.5 MPa, respectively. Owing to its excellent bonding ability and long-term stability, cement is widely used in roadbed reinforcement and soft soil stabilization. During hydration, cement forms products such as C-S-H gel and ettringite, which significantly enhance the strength and durability of the soil. The lime was purchased from Shanshui Chemical Co., Ltd, Hubei, China. and complies with the standard Construction Quicklime (JC/T 479-2013) [23]. It contains CaO ≥ 85% and MgO ≤ 5%, with a fineness of 80 mesh. As a commonly used alkaline stabilizing material, lime produces calcium hydroxide through ion exchange and pozzolanic reactions, thereby improving the mechanical properties and strength of the soil. The solid waste-based cementitious material was prepared according to an optimized proportion derived from previous studies, using alkali slag (SR), slag (SL), and phosphogypsum (PY) as raw materials with mass fractions of 10%, 81%, and 9%, respectively. All raw materials were purchased from Kangheng Environmental Company, Shanghai, China, and blended after grinding and processing. This material belongs to a hydraulic cementitious system designed for the resource utilization of industrial solid waste. During hydration, products such as C-S-H gel and ettringite are formed, which enhance the mechanical properties and durability of the soil. The microbial curing agent used in this study was *Sporosarcina pasteurii* (ATCC 11859; formerly *Bacillus pasteurii*), purchased from Beijing Baocang Biotechnology Co., Ltd, Beijing, China [24]. The culture medium consisted of 15 g casein peptone, 5 g soy peptone, 20 g agar, 5 g sodium chloride, and 900 mL double-distilled water. Sodium chloride was used to regulate microbial osmotic pressure, while urea served as the carbon source for biomineralization. Microbial curing agents reinforce soil through microbial-induced calcium carbonate precipitation (MICP), and the reaction can proceed under ambient temperature and pressure. Calcium carbonate crystals formed during microbial mineralization fill soil pores and bond soil particles, thereby enhancing the strength and long-term stability of the soil. Considering the reinforcement efficiency, environmental impact, and economic performance of the four curing agents, comparative experiments were conducted to investigate their modification effects and mechanisms in TSC composite soil, providing a theoretical basis and technical support for road engineering and related soil stabilization applications.

### 2.2. Experimental Program

**Figure 2:**
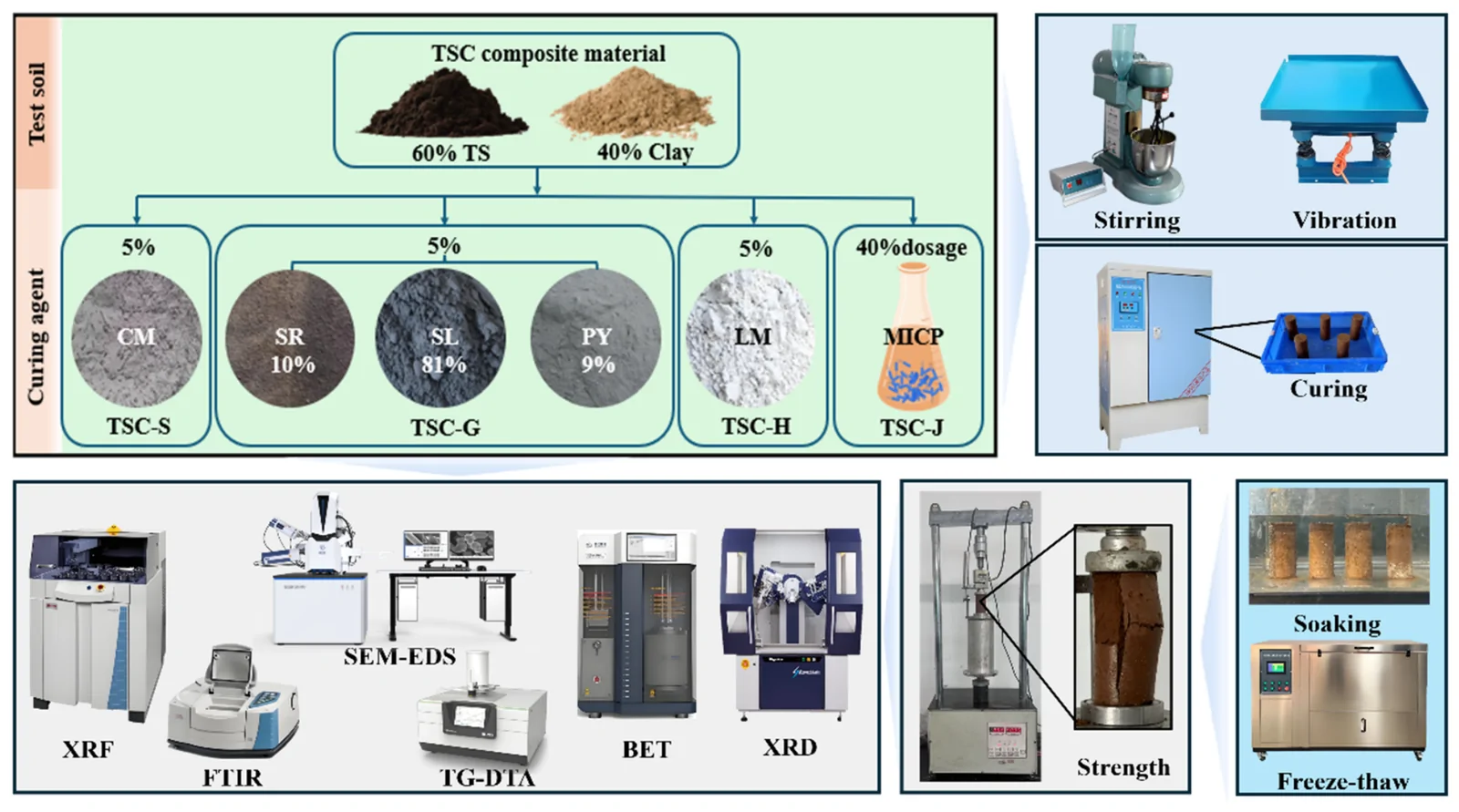
Experimental flowchart.

In this study, TSC composite soil was used as the base material, and comparative experiments were conducted using four curing methods: cement, lime, solid waste-based cementitious materials, and microbial curing agents. The aim was to investigate the effects of different curing agents on the mechanical properties, microstructural characteristics, and carbon emissions of TSC composite soil, and to identify suitable curing schemes. The dry density of the base material was controlled at 1427.03 kg·m^−3^ to ensure consistent initial conditions and basic physical properties of the TSC composite soil samples. All samples were cured in a standard curing chamber at a temperature of 20 ± 2 °C and a relative humidity of 65 ± 5%. Four curing ages (3, 7, 14, and 28 days) were selected to evaluate the evolution of mechanical properties at different stages. For each test group, three parallel specimens were prepared, and the results are reported as mean ± standard deviation (SD). The test groups for different curing systems are presented in Table 3. Among them, TSC-S, TSC-G, TSC-H, and TSC-J represent cement-cured specimens, solid waste-based cementitious material-cured specimens, lime-cured specimens, and microbially cured specimens, respectively. The curing tests using cement, lime, and solid waste-based cementitious materials were designed based on preliminary experiments, and the dosage was determined to be 5% by dry mass of TSC composite soil. During sample preparation, the raw materials were weighed according to the designed proportions and dry-mixed for 2 min to ensure uniform distribution. Distilled water was then added to adjust the moisture content to the optimum value of 25%, which was determined from the liquid and plastic limits of the TSC composite soil. The mixture was further stirred for 3 min to obtain a homogeneous mixture. The mixture was subsequently placed into a cylindrical mold (Φ 80 mm × 39.1 mm) in layers, and each layer was compacted by 20 vibrations to ensure adequate density. After standing for 48 h, the specimens were demolded and cured under standard conditions until the specified age. *Bacillus pasteurii* was selected as the functional strain for the microbial curing test. The bacterial suspension was used when the optical density at 600 nm (OD_600_) exceeded 1.5. Urea was used as the carbon source, and calcium chloride was used as the calcium source (Ca^2+^). Based on preliminary experiments, the bacterial suspension dosage was determined to be 40% of the total water required to achieve the target moisture content of 25% in the TSC composite soil. Accordingly, the bacterial suspension accounted for 40% of the total water addition, while the remaining water requirement was met using distilled water. For the microbial treatment group, the bacterial suspension was first mixed with the TSC composite soil. The urea and calcium chloride solutions were subsequently added to the mixture. The resulting mixture was then placed into the cylindrical mold (Φ 80 mm × 39.1 mm) and compacted according to the specimen preparation procedure described above. The specimens were subsequently cured under the specified standard conditions until the designated testing ages. Unconfined compressive strength, water stability, freeze–thaw resistance, and microstructural tests were conducted to systematically analyze the effects of the four curing agents on the mechanical properties and structural characteristics of TSC composite soil at different curing ages. The overall experimental procedure is illustrated in Figure 2.

### 2.3. Testing Methods

#### 2.3.1. Basic Property Tests

This study conducted a comprehensive evaluation of the basic properties of TSC composite soil, including unconfined compressive strength, water stability, and freeze–thaw resistance.

##### Unconfined Compressive Strength Test

The unconfined compressive strength (UCS) test was conducted in accordance with the Standard for Geotechnical Test Methods (GB/T 50123-2019) [25]. Cylindrical specimens (Φ 80 mm × 39.1 mm) were prepared using the static compaction method, and the height-to-diameter ratio was maintained at greater than 2 to ensure uniform stress distribution and prevent eccentric compression during testing. After preparation, the specimens were immediately placed in a standard curing chamber, where the curing conditions were maintained at a temperature of 20 ± 2 °C and a relative humidity of ≥60%. During curing, the specimens were protected from disturbance and moisture loss to ensure normal strength development. After reaching the designated curing age, the specimens were removed and both ends were polished with sandpaper to ensure that the surfaces were flat and perpendicular to the specimen axis. The specimen was then placed at the center of the loading plate of the automatic unconfined compressive strength testing machine, and the upper loading plate was adjusted to gently contact the specimen surface to avoid damage caused by excessive initial load. The test was conducted under displacement-controlled loading with a rate of 1 mm/min. The load and corresponding displacement were recorded continuously until specimen failure and the peak load was reached, after which loading was terminated. The unconfined compressive strength was calculated based on the peak load. Three parallel specimens were tested for each group, and the mean and standard deviation were calculated from the measured values. This test quantitatively evaluates the effects of curing agent dosage and curing age on the mechanical properties of TSC composite soil.

##### Water Stability Test

The water stability test was conducted in accordance with JTG 3441-2024 Test Method for Subgrade Fill Materials [26] to evaluate the stability of the mechanical properties of TSC composite soil under immersion conditions and to provide a basis for its engineering application in humid or groundwater-rich areas. Two groups of cylindrical specimens with identical dimensions were prepared, following the same molding method and standard curing conditions as those used in the unconfined compressive strength (UCS) test. One group of specimens was tested for UCS after curing to the designated age *T*, and the measured strength was recorded as the reference strength *S*. The other group of specimens was cured to age *T* − 1. The specimens were then removed and wrapped with plastic film along the sides, leaving only the top and bottom surfaces exposed to prevent lateral water infiltration. The specimens were subsequently immersed vertically in pure water at 20 ± 2 °C. During soaking, the water level was maintained at least 20 mm above the top surface of the specimens. The soaking duration was controlled at 1 day, during which water was replenished regularly to maintain a constant water level. After immersion, the specimens were removed, surface moisture was dried with filter paper, and the UCS test was immediately conducted at the same loading rate as the reference group. The resulting strength was recorded as the immersed strength *S_w_*. To quantitatively evaluate water stability, the water stability coefficient *K* was calculated as follows:

*K* = *S_w_*/*S* (1)

Here, *S_w_* represents the UCS (kPa) measured after 1 day of immersion following curing to age *T* − 1, while *S* represents the UCS (kPa) measured after curing to age *T*. Three parallel specimens were prepared for each test group. The values of *S*, *S_w_*, and the water stability coefficient *K* were calculated for each group of specimens. Data with excessive dispersion (deviation > 15%) were excluded, and the average value was used as the final result. Generally, a larger water stability coefficient *K* indicates stronger resistance to water erosion and better water stability of the TSC composite soil. When *K* ≥ 0.7, the material satisfies the water stability requirements for general subgrade fill materials. This index was used to evaluate the improvement effect of curing agents on the water stability of TSC composite soil.

##### Freeze–Thaw Cycle Test

The freeze–thaw cycle test was conducted in accordance with the Standard for Geotechnical Test Methods (GB/T 50123-2019) [27] and ASTM D5918-2013 [28], under the same standard curing conditions as those described above. The test aimed to evaluate the frost resistance and long-term stability of cured TSC composite soil, providing data support for its application in subgrade engineering in cold regions subjected to freeze–thaw cycles. After curing to the designated age, the specimens were first subjected to vacuum saturation to eliminate the influence of initial moisture content differences and then placed in a programmable freeze–thaw chamber for testing. The test employed an alternating freeze–thaw cycle mode. During the freezing stage, the temperature was maintained at −18 ± 2 °C for 12 h to ensure complete freezing of the specimens. During the thawing stage, the specimens were immersed in a water bath at 20 ± 2 °C for 12 h to ensure uniform thawing. Each freeze–thaw cycle lasted 24 h, and a total of eight cycles were applied in this test. The test was paused after every two cycles. A precision electronic balance was used to measure mass changes, and a universal testing machine was used to determine the attenuation of unconfined compressive strength. In addition, visual changes such as surface spalling and crack development were recorded, and the mass loss rate and strength attenuation rate were calculated. The termination criteria were defined as follows: the test was stopped when the mass loss rate reached 5% or when the relative dynamic elastic modulus decreased to 60% of its initial value, regardless of whether the preset number of cycles had been completed. This test enables systematic analysis of the deterioration mechanisms of microstructure and macroscopic mechanical properties of TSC composite soil under freeze–thaw cycles and provides a comprehensive evaluation of its frost resistance durability and long-term structural stability.

#### 2.3.2. Microscopic Testing Methods

##### Phase Analysis Method for the Products

In this study, an X-ray diffractometer (TD-3500) [29] was employed to identify the crystalline phases of TS, TSC, and TSC-J. Measurements were conducted using Cu Kα radiation with a scanning range of 5–85° and a scanning rate of 0.3° s^−1^. In contrast to the crystalline phases identified by XRD, functional groups in the samples were further analyzed using a Fourier transform infrared spectrometer (FTIR-650S) for complementary characterization [30]. The spectra were obtained using the potassium bromide (KBr) pellet method. The scanning procedure included background acquisition and sample measurement, with a total of 64 scans. The instrument resolution and aperture ratio were set to 2.0 cm^−1^ and 0.625, respectively.

##### Product Quantity Relationship Testing Method

The carbon sequestration capacity was determined using a thermogravimetric analyzer (HTG-2) [31]. Approximately 10 mg of the sample was weighed and placed in an alumina crucible. The sample was then heated from room temperature to 1000 °C at a heating rate of 10 °C min^−1^. The mass loss of the sample at different temperatures was recorded using a high-precision electronic balance.

##### Testing Methods for Microstructural Characteristics

The microstructural morphology of TS, TSC, and TSC-J was observed using a COXEM EM-30 scanning electron microscope (SEM) [32], and the elemental composition was analyzed using a QUANTAX XFlash energy-dispersive spectrometer (EDS, Bruker Nano GmbH, Berlin, Germany) [33]. Due to the poor electrical conductivity of the samples, they were coated with a thin gold layer by ion sputtering prior to testing. After the samples were mounted on the specimen stage and evacuated to a high vacuum, an appropriate accelerating voltage and working distance were selected for imaging. When necessary, EDS point or area spectra were simultaneously collected. In addition, the specific surface area and pore structure were determined using the nitrogen adsorption–desorption method (BET) [34]. To prevent structural changes in gypsum hydrates and MICP products, the powder samples were pretreated by vacuum degassing to remove physically adsorbed water and volatile substances. The measurements were conducted at 77 K (liquid nitrogen), and adsorption–desorption isotherms within a relative pressure range of P/P_0_ = 0.01–0.99 were recorded for comparison and cross-validation with the morphological features observed by SEM/EDS.

## 3. Results and Discussion

### 3.1. Evaluation of Mechanical Performance and Environmental Durability

#### 3.1.1. Analysis of Unconfined Compressive Strength

**Figure 3:**
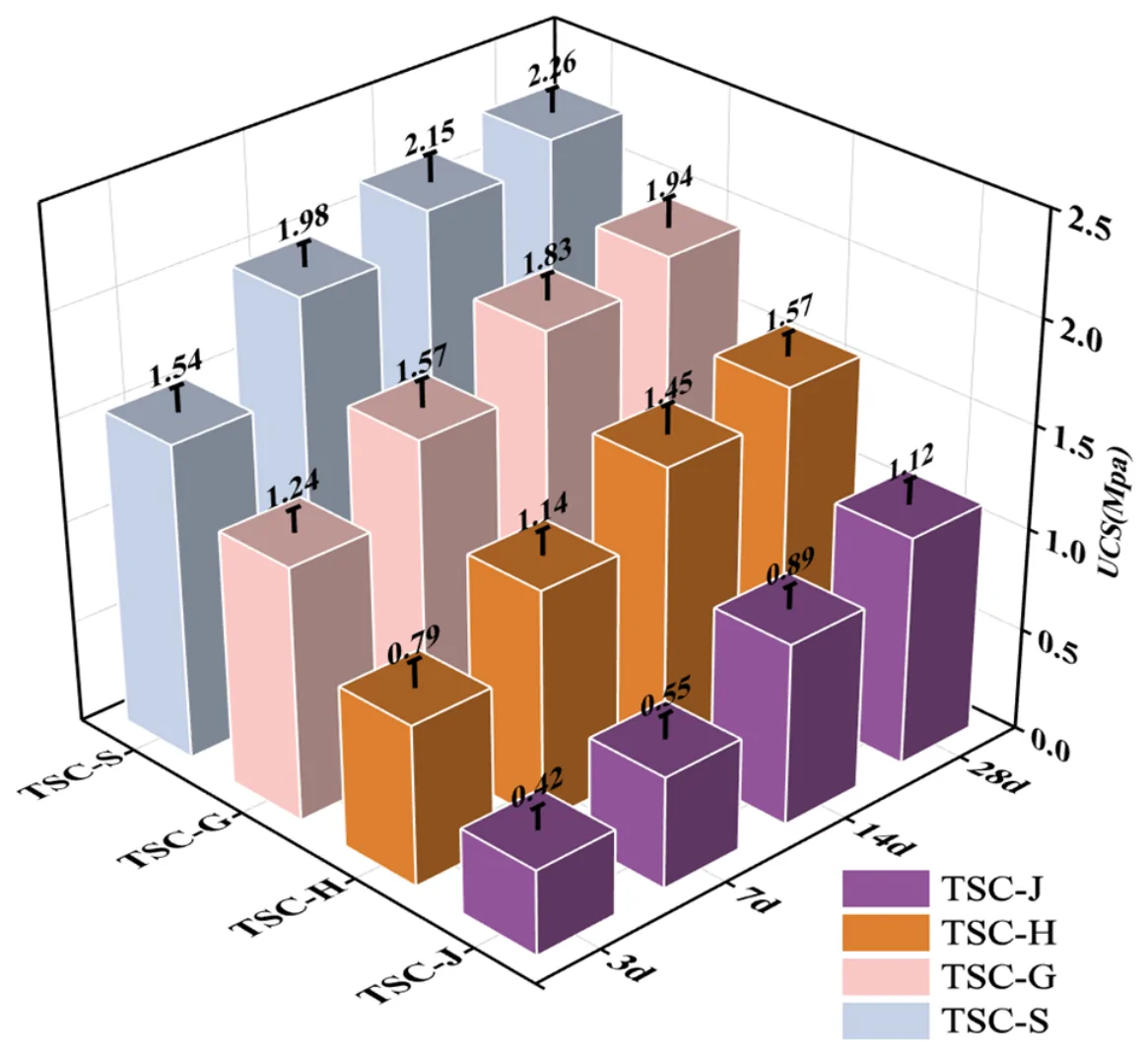
Data graph of UCS with different curing methods.

Figure 3 illustrates the evolution of unconfined compressive strength (UCS) of TSC composite soil with curing age under various curing methods. Overall, the UCS of samples treated with 5% cement, 5% lime, 5% solid waste-based cementitious materials, and 40% microbial content exhibited a continuous increase with curing age, suggesting that these curing methods can enhance the structural integrity and load-bearing capacity of the TSC composite soil system. This trend is consistent with previous observations in industrial by-product gypsum-based materials, where ongoing reactions gradually densify the pore structure and promote strength development [35]. In terms of relative strength, the ranking among different curing methods remained consistent across all ages: cement-cured samples exhibited the highest UCS, followed by solid waste-based and lime-cured samples, while microbial curing resulted in the lowest strength.

The TSC-S system exhibited a pronounced early strength advantage, achieving an approximate UCS of 2.6 MPa at 28 days, markedly higher than that observed for other curing methods. This behavior is primarily attributed to the formation of abundant C-S-H gel during cement hydration, which establishes a continuous cementing network within the sample. Additionally, the abundant SO_4_^2−^ in the TSC composite soil facilitates the formation of ettringite (AFt), contributing to the early development of a relatively stable “gel-crystal” composite framework, which substantially enhances the material’s load-bearing capacity [36]. The TSC-G system generally exhibits intermediate UCS, with a 28-day strength of approximately 1.94 MPa. Compared with cement curing, its early strength is slightly lower; however, the growth during the middle and later stages is relatively continuous, indicating promising development potential. This system represents a typical alkali-activated mineral solid waste cementitious material. The highly alkaline environment supplied by the alkali residue effectively activates latent Si and Al in the slag, producing cementitious products predominantly composed of C-(A)-S-H gels [37]. The addition of a small amount of phosphogypsum not only supplements SO_4_^2−^ but also promotes AFt formation, thereby jointly enhancing the structural density and mechanical properties of the system [38]. However, owing to a relatively slower activation reaction rate compared with the cement system, early strength development in TSC-G is somewhat limited. This observation agrees with recent research on ternary binders entirely composed of industrial by-products, which demonstrated that waste-based binders can simultaneously improve soil mechanical performance and reduce environmental burdens as sustainable substitutes for ordinary Portland cement. The TSC-H system exhibits lower strength compared with the cement- and solid waste-based systems. Its 28-day UCS is approximately 1.57 MPa, with a relatively moderate strength growth rate. Lime-induced solidification primarily relies on the alkaline environment provided by Ca(OH)_2_, which activates pozzolanic reactions of active silica and alumina in the clay, producing a certain amount of C-S-H and C-A-H gels [39]. However, at relatively high TSC composite soil content, the availability of reactive Si and Al for the reaction is limited. Furthermore, lime lacks intrinsic cementitious capability, limiting both the quantity and density of the formed cementitious products, and consequently restricting its mechanical strengthening effect [40]. Such relatively moderate improvement performance is consistent with modern experimental benchmarks for lime-treated soils, in which hydrated lime alters shear strength, compressibility and consolidation behaviours but shows performance constraints under complex soil matrix conditions [41]. The TSC-J system displayed the lowest UCS across all curing ages. Its 28-day strength was approximately 1.12 MPa, yet UCS continued to increase with curing age. This system relies primarily on microbiologically induced calcium carbonate precipitation (MICP). Microorganisms secrete urease, catalyzing urea hydrolysis to generate carbonate ions (CO_3_^2−^), which react with environmental calcium ions (Ca^2+^) to precipitate calcium carbonate, filling pores, cementing particles, and forming a stable structure [42]. However, in the TSC composite soil system, elevated SO_4_^2−^ concentrations and the complex chemical environment of the pore solution may adversely affect microbial activity and the uniformity of calcium carbonate deposition, hindering the formation of a continuous and dense cementing network [43]. Consequently, its short-term mechanical performance is markedly inferior to that of traditional cement-based curing systems.

A comprehensive analysis indicates that, within the TSC composite soil system, cement curing exhibits the greatest advantages in mechanical performance. Solid waste-based solidification not only achieves high strength but also offers potential for resource recovery and sustainable development. The effectiveness of lime curing is constrained by the system composition. Although microbial curing exhibits relatively low mechanical performance, it presents a valuable avenue for future research on durability and environmentally responsive curing methods.

#### 3.1.2. Investigation of Water Stability

**Figure 4:**
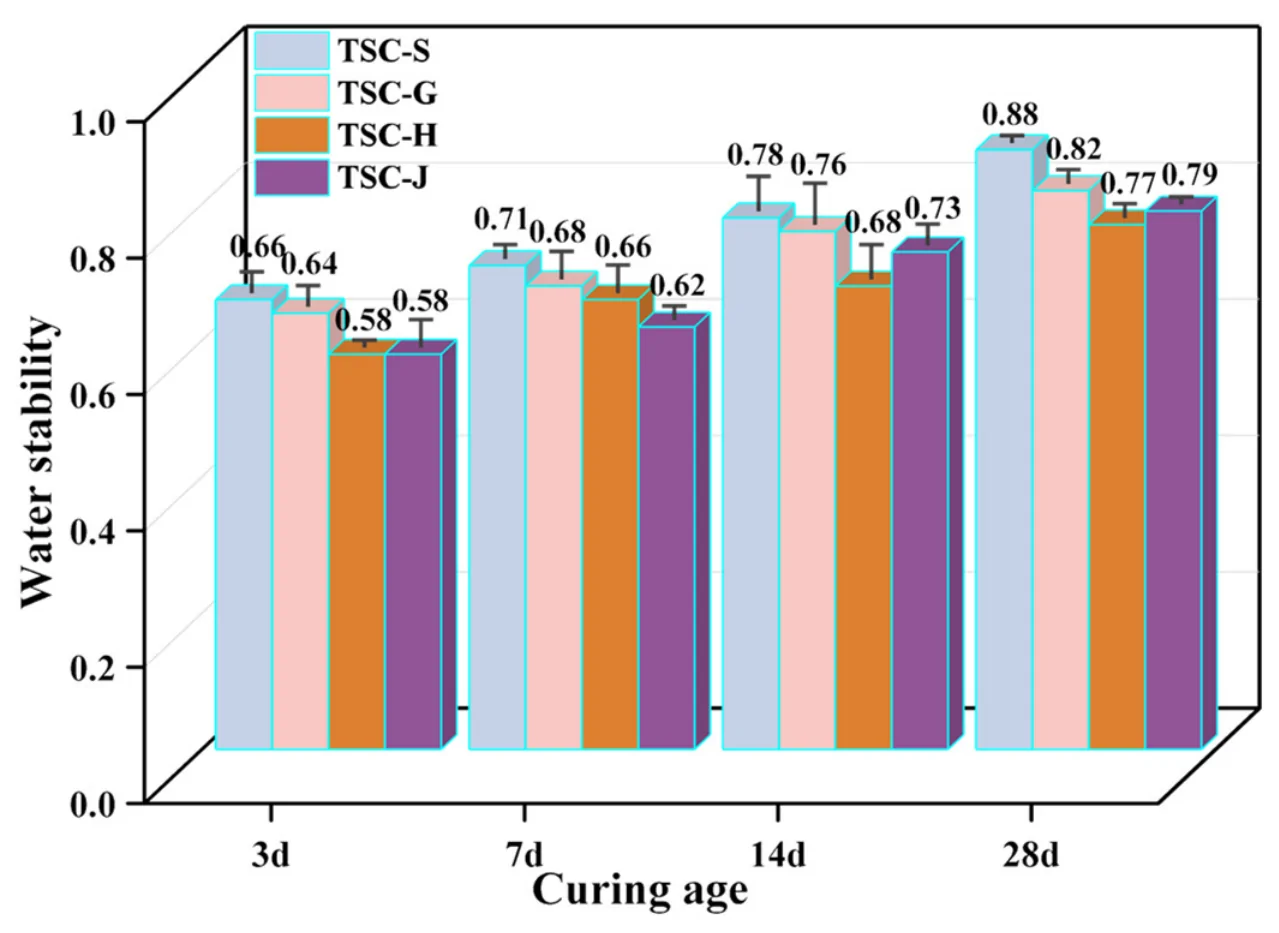
Water stability data graph of different curing methods.

Regarding water stability, TSC composite soil exhibits a distinct evolution pattern under different curing methods with increasing curing age, as illustrated in Figure 4. Overall, the water stability indicators of all four curing systems continuously increased with curing age. No significant fluctuations or decreases were observed from 3 to 28 days, indicating that, as the curing reactions progressed, the internal structure gradually densified, and water-induced structural integrity continuously improved. These findings are consistent with the previously observed trend of UCS increasing with age, highlighting a synergistic relationship between strength development and the enhancement of water stability. Comparative analysis of different curing methods shows that the TSC-S system exhibits the highest water stability across all curing ages, increasing from 0.66 at 3 days to 0.88 at 28 days, consistently maintaining a dominant position. This superior performance is primarily attributed to the abundant C-S-H gel and stable hydration products, such as ettringite, generated during cement hydration. These products form a continuous, dense, and highly water-resistant cementation network between particles, effectively inhibiting structural softening and particle detachment induced by water infiltration [44]. Therefore, cement curing not only excels in mechanical performance but also markedly enhances water resistance and stability of the material. The TSC-G system generally exhibits intermediate water stability, with a 28-day value of approximately 0.82, slightly lower than cement curing but considerably higher than lime and microbial curing systems. The continuous enhancement of water stability in this system is primarily related to the progressive development of the alkali-activated slag reaction. The progressive formation of cementitious gels helps fill pores and strengthen contacts between soil particles, thereby reducing the susceptibility of the soil structure to water-induced weakening. As the curing period extends, the C-(A)-S-H gel formed in the system progressively fills pores and encapsulates particles. Additionally, a small amount of sulfate introduced via phosphogypsum participates in the reaction, contributing to the formation of a relatively stable cementitious structure and enhancing the material’s structural integrity in aqueous environments [45]. However, compared with the cement system, differences in hydration product types and reaction rates result in slightly lower water stability. The TSC-H system generally exhibits low water stability, with values increasing from 0.58 at 3 days to 0.77 at 28 days, reflecting a relatively limited growth rate. This behavior is primarily associated with the lime-based curing mechanism. Lime itself lacks intrinsic cementitious capability, and improvements in water stability rely mainly on pozzolanic reactions involving reactive silica and alumina in the clay [46]. However, at high titanium gypsum dosages, the availability of reactive silica and alumina is limited, resulting in a small and discontinuously distributed amount of cementitious products. Consequently, the material remains susceptible to localized softening and structural weakening under water exposure [47], limiting further enhancement of water stability. The TSC-J system exhibits slightly lower early water stability compared with cement- and solid waste-based systems, though the difference relative to lime curing is minimal. Its water stability increased from 0.58 at 3 days to 0.79 at 28 days, exhibiting a relatively stable growth trend. Microbial curing functions primarily by inducing calcium carbonate precipitation (MICP) through microorganisms, forming bridging structures at particle contacts and thereby enhancing resistance to water erosion to some extent [48]. However, within the TSC composite soil system, elevated sulfate concentrations and the chemical environment of the pore solution may adversely affect microbial activity and the continuity of calcium carbonate deposition, resulting in localized CaCO_3_ formation and impeding the development of a continuous, dense, water-resistant cementation network [49]. Consequently, improvements in water stability remain relatively limited.

Overall, the water stability of TSC composite soil under different curing methods closely follows the trend of unconfined compressive strength development, indicating that the extent of internal cementation formation is a key factor governing both strength and water stability. Cement curing exhibits the highest water stability. Solid waste-based curing achieves high water stability while offering advantages in resource utilization, whereas lime- and microbial-based curing systems exhibit certain limitations under high dosage conditions. These findings provide an important comparative basis for subsequent freeze–thaw and long-term durability assessments.

#### 3.1.3. Evaluation of Freeze–Thaw Resistance

**Figure 5:**
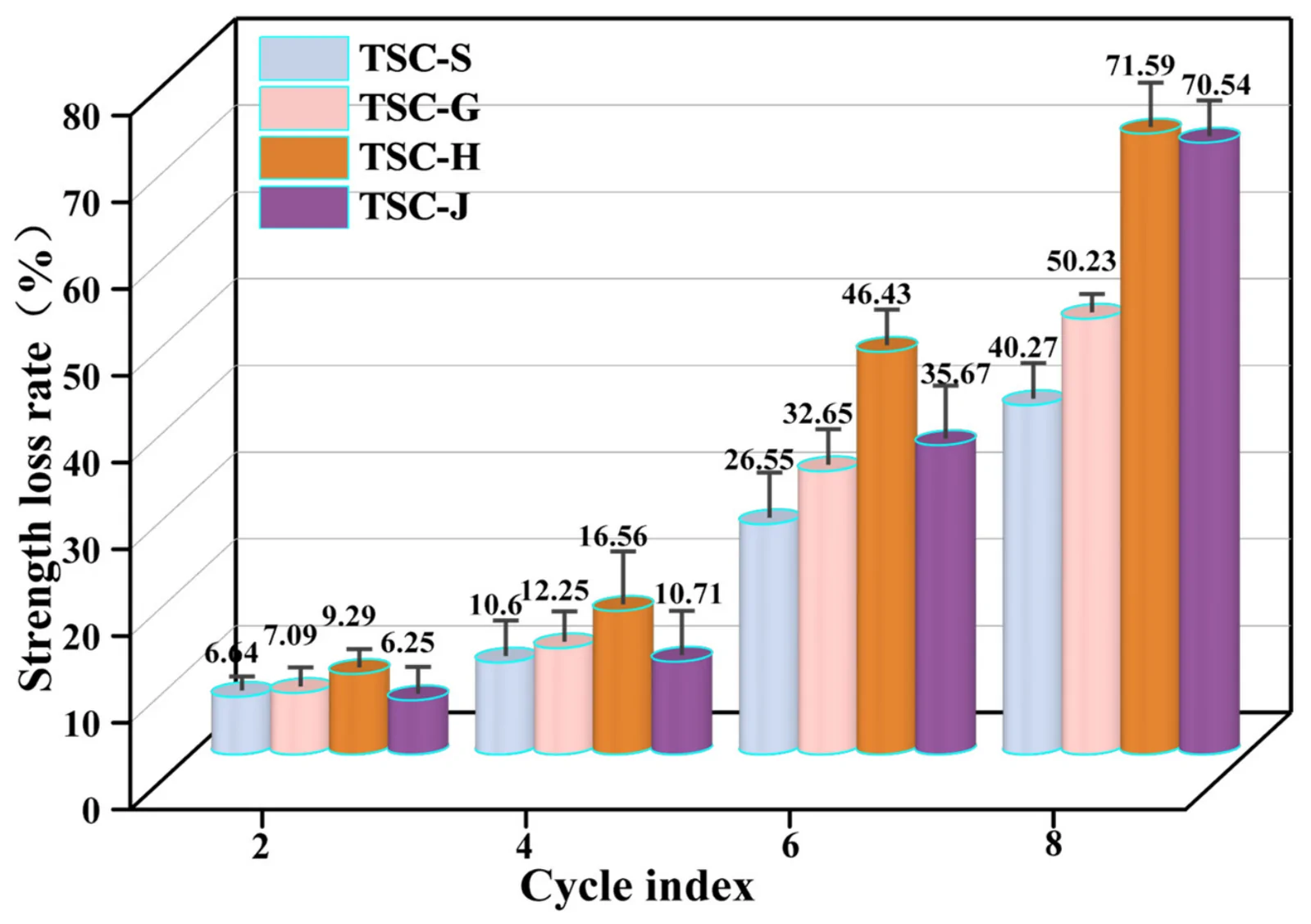
Freeze–thaw cycle data graph of different curing methods.

Figure 5 illustrates the variation in unconfined compressive strength (UCS) of TSC composite soil systems subjected to freeze–thaw cycles under different curing methods. With increasing freeze–thaw cycles, the UCS of all samples exhibited a continuous decline, indicating that freeze–thaw action significantly deteriorates the structure of the solidified soil [50]. Prior to freeze–thaw cycling, substantial differences in initial UCS were observed among the curing systems. The initial UCS of cement-cured samples reached 2.26 MPa, indicating that a relatively complete cementitious skeleton had formed through the generation of abundant hydration products [9]. In contrast, microbial-cured samples exhibited the lowest initial strength, at only 1.12 MPa. The curing effect primarily relies on CaCO_3_ precipitation, with a relatively limited diversity of cementation phases [51]. As the number of freeze–thaw cycles increased to 2–4, the UCS of each sample declined to varying extents. Notably, the strength retention of cement- and solid waste-based curing systems was considerably higher than that of lime- and microbial-based systems. After four freeze–thaw cycles, the UCS of cement- and solid waste-based cured samples remained at 2.02 MPa and 1.72 MPa, respectively, whereas the UCS of microbial-cured samples decreased to approximately 1.00 MPa. This indicates that hydration products, such as AFt and C-S-H, formed in cement- and solid waste-based systems can partially resist structural damage induced by ice crystal expansion during freeze–thaw cycles [52]. The relatively continuous cementitious network may help maintain interparticle bonding during repeated freezing and thawing, whereas less continuous cementation may provide insufficient resistance to ice-induced expansion and thawing-related structural damage. These interpretations are further supported by previous MICP investigations, which highlighted that MICP-produced calcium carbonate precipitates can enhance soil cohesion and water-resistant capacity via pore filling and particle bridging, yet durability performance strongly depends on the continuity and morphology of carbonate crystals under cyclic environmental loadings [53]. As the number of freeze–thaw cycles increases to 6–10, strength degradation in all curing systems intensifies, particularly in lime- and microbial-cured samples, which exhibit the most pronounced UCS reduction. After 10 freeze–thaw cycles, the UCS of microbial-cured samples nearly approached zero, indicating severe damage to the internal cementation structure and substantial enhancement of pore connectivity under repeated freeze–thaw action [54]. In contrast, cement-cured samples retained a strength of approximately 1.01 MPa after 10 cycles, whereas solid waste-based cured samples remained at 0.71 MPa, demonstrating superior freeze–thaw durability.

Comprehensive analysis indicates that freeze–thaw cycling progressively weakens the bonding between cementitious phases and soil particles within the solidified soil due to repeated freezing expansion and thawing contraction [55]. Cement- and solid waste-based curing systems, owing to the formation of a multiphase, continuous, and interwoven network of hydration products, exhibit substantially superior structural stability and resistance to freeze–thaw damage compared with lime- and microbial-based systems [52]. These findings are consistent with the aforementioned XRD, TG-DTG, and BET pore structure analyses, further corroborating the decisive influence of curing mechanisms on the evolution of macroscopic mechanical properties.

It should be emphasized that the pronounced strength deterioration of MICP-treated TSC composite soil under repeated freeze–thaw cycles represents a significant limitation for its practical engineering application. Although MICP treatment offers potential environmental benefits through reduced carbon emissions, the nearly complete loss of compressive strength after 10 freeze–thaw cycles indicates insufficient durability under severe freezing and thawing conditions. This deterioration may be attributed to the progressive disruption of calcium carbonate cementation bonds and the development of interconnected pores caused by repeated frost expansion and thawing. Consequently, the direct application of MICP-treated TSC composite soil in cold regions subjected to frequent freeze–thaw cycles is not recommended without further durability enhancement. Future research should focus on optimizing calcium carbonate precipitation, improving the continuity of interparticle cementation, and evaluating long-term freeze–thaw performance under field-representative conditions.

### 3.2. Analysis of Micro-Mechanisms

#### 3.2.1. Phase Analysis of Products

The effects of various curing methods on the microstructure of TSC composite soil and the formation of hydration and cementation products were systematically investigated using three analytical techniques: SEM-EDS, FTIR, and XRD. By integrating the triple evidence chain of XRD crystal phases, FTIR functional groups, and SEM-EDS morphology and elemental composition, the results clearly demonstrate that different curing agents induce significant alterations in soil structure, promote the formation of diverse cementation and secondary mineral phases, and enhance the compactness and strength of the soil to a certain extent.

**Figure 6:**
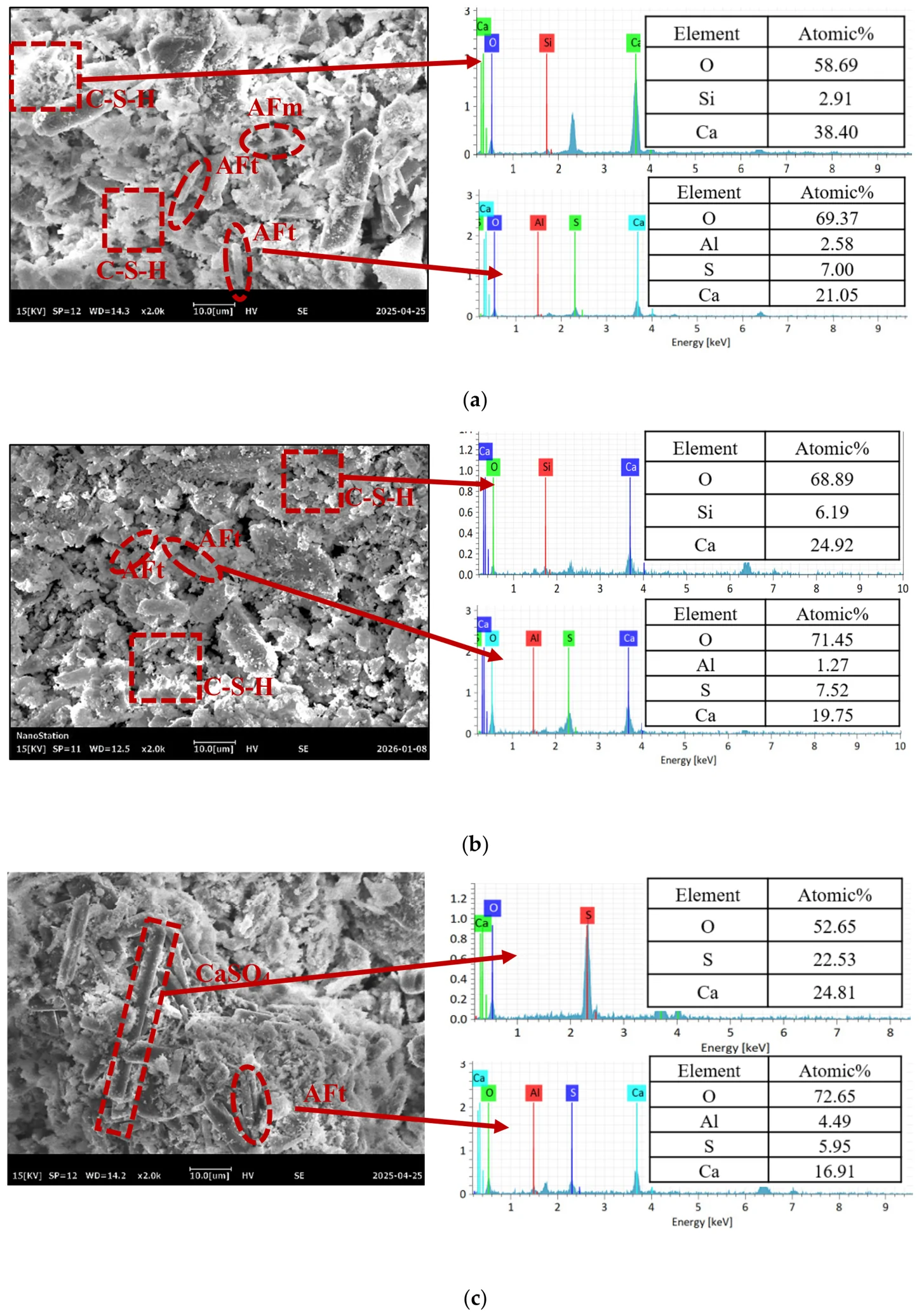

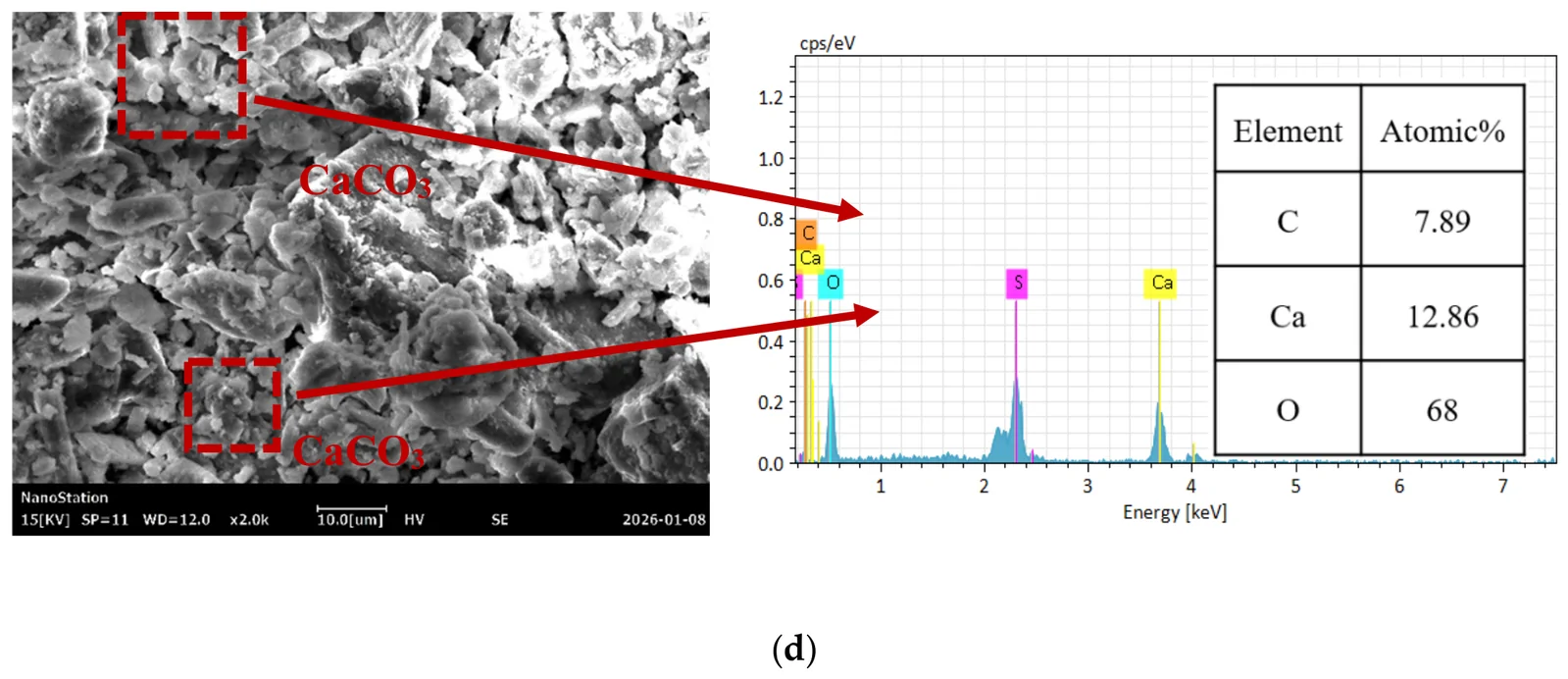
SEM and EDS spectra of different curing methods: (**a**) SEM and EDS spectra of TSC-S; (**b**) SEM and EDS spectra of TSC-G; (**c**) SEM and EDS spectra of TSC-H; (**d**) SEM and EDS spectra of TSC-J.

**Figure 7:**
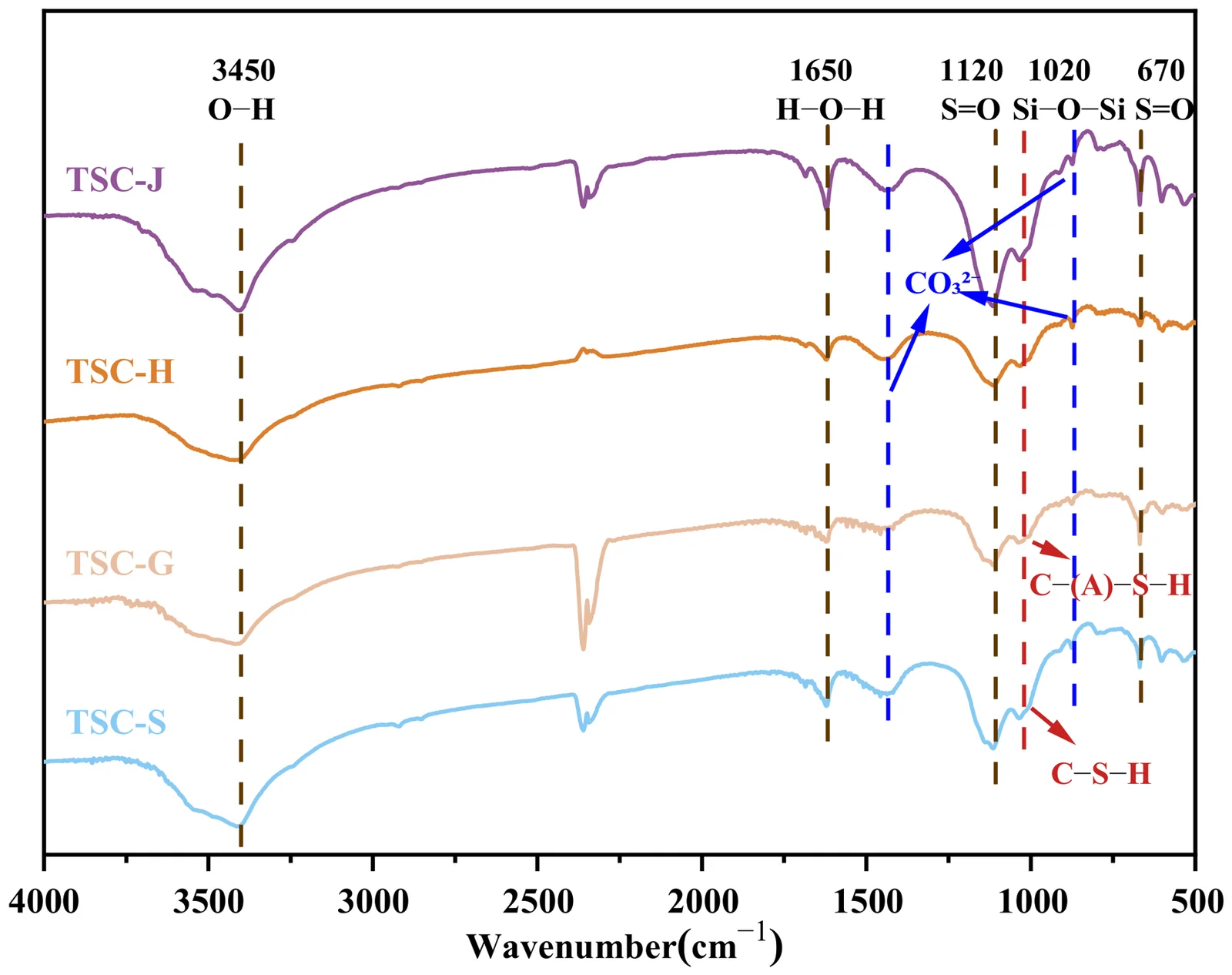
The 28d FTIR patterns of different curing methods.

**Figure 8:**
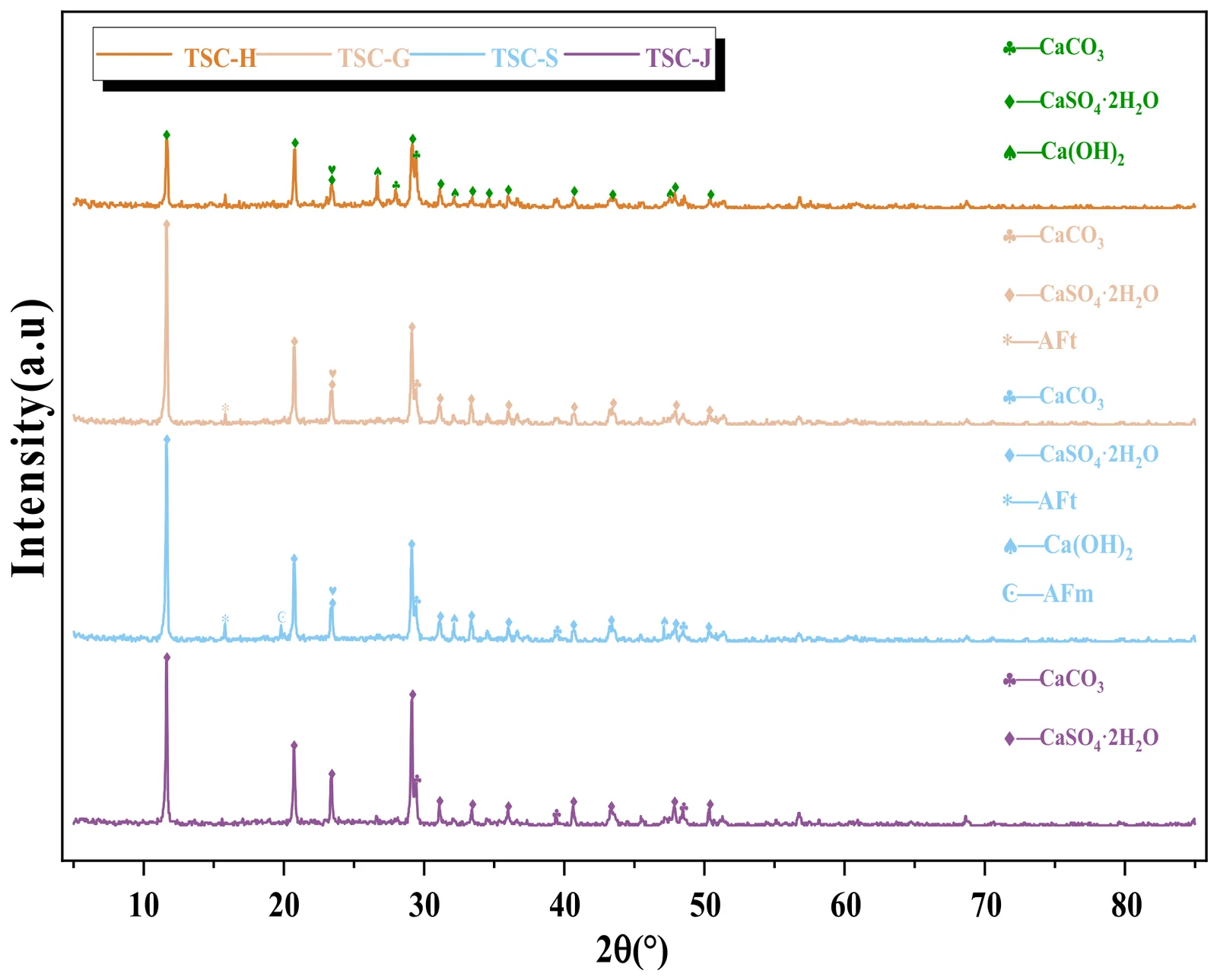
The 28d XRD patterns of different curing methods.

Figure 6a shows that in the cement-cured (TSC-S) system, in addition to CaCO_3_ and CaSO_4_·2H_2_O, AFt and AFm hydration products were also detected, indicating that cement hydration continued over 28 days, producing C-S-H gel and sulfate minerals. These hydration products occupy soil pores and enhance interparticle cementation [56]. Figure 7 further confirms the formation of C-S-H gel. The intensified peaks at 3450 cm^−1^ and 1650 cm^−1^ indicate an increase in bound water within the hydration products, while the enhanced characteristic peaks of Si-O-Si and Si-O-Al at 1020 cm^−1^ confirm the formation of silicoaluminate gel [57]. SEM-EDS results (Figure 6a) indicate that cement hydration products are widely distributed on particle surfaces, forming a dense cementation network that effectively enhances soil strength and stability [9]. In the solid waste-based cementitious (TSC-G) system, the XRD pattern (Figure 8) shows that, apart from CaCO_3_ and CaSO_4_·2H_2_O, the AFt crystal peak is relatively weak, and no distinct Ca(OH)_2_ peak is observed. This indicates that the solid waste-based system primarily forms low-crystallinity, silica-rich aluminum gels (e.g., C-(A)-S-H and N-A-S-H) via alkali-activated reactions [58]. Figure 7 exhibits enhanced absorption peaks of Si-O-Si and Si-O-Al, further confirming the formation of silicoaluminate gel [59]. SEM-EDS analysis (Figure 6b) demonstrates that the gel products are relatively uniformly distributed among particles, effectively occupying pores and promoting interparticle cementation [60]. The strengthening mechanism of the solid waste-based solidification (TSC-G) system is primarily governed by gels formed via alkali-activated reactions. These gel products can contribute to strength development by binding soil particles and filling pores. This interpretation is consistent with the observed microstructural characteristics, although the individual contribution of each reaction product cannot be quantified from the present characterization results alone. This cementation enhances soil compactness and mechanical properties. This cementation enhances soil compactness and mechanical properties. Similar mineral-phase evolution behaviours have been reported for waste-derived binder systems consisting of oil-shale ash and siliceous pozzolan additives, where promoted C-S-H generation and microstructure densification effectively mitigated the engineering drawbacks of problematic soils, offering low-cost alternative stabilization routes for waste resource recycling [61]. The lime-cured (TSC-H) system exhibits distinct curing mechanisms. XRD results (Figure 8) indicate that some Ca(OH)_2_ remains in lime-solidified soil, whereas CaCO_3_ forms via carbonation, suggesting that the lime-solidified system undergoes three sequential stages: flocculation-agglomeration, pozzolanic reaction, and carbonation deposition [62]. The distinct CaCO_3_ absorption peak in Figure 7 indicates that carbonation significantly contributes to the enhancement of soil structure [63]. SEM-EDS results (Figure 6c) reveal that pores in the lime-cured system are relatively prominent and the cementing phase is dispersed, indicating incomplete pore filling and a loosely formed cementing network, which limits the strength enhancement [64]. The microbial-cured (TSC-J) system primarily relies on microbially induced calcium carbonate precipitation (MICP) for reinforcement. In the XRD pattern (Figure 8), the CaCO_3_ diffraction peak is pronounced, whereas AFt, AFm, and Ca(OH)_2_ peaks are relatively weak, indicating that the microbial-solidified soil primarily forms the cementing phase via CaCO_3_ precipitation [65]. The characteristic CO_3_^2−^ absorption peak in Figure 7 is significantly intensified at 1400–1500 cm^−1^, indicating that microbial metabolism facilitates CaCO_3_ formation [66]. SEM-EDS results (Figure 6d) reveal a distinct bridging structure between particles in the microbially stabilized soil. CaCO_3_ crystals occupy pores and strengthen interparticle connections, markedly improving soil structural integrity and strength [67].

By integrating SEM-EDS, FTIR, and XRD analyses, the curing mechanisms of TSC composite soil under various treatment methods were comprehensively elucidated. Cement-cured and solid waste-based systems generate gel products via hydration and alkali-activated reactions, promoting interparticle cementation and pore filling. In contrast, lime-cured soil primarily strengthens the structure through carbonation reactions and limited cementitious phases. Microbial-cured soil is reinforced through microbially induced CaCO_3_ precipitation. The reinforcing effects of these curing strategies differ in mechanism and emphasis, yet all enhance soil stability and strength through pore filling and interparticle cementation.

#### 3.2.2. Quantitative Relationship of Curing Products

**Figure 9:**
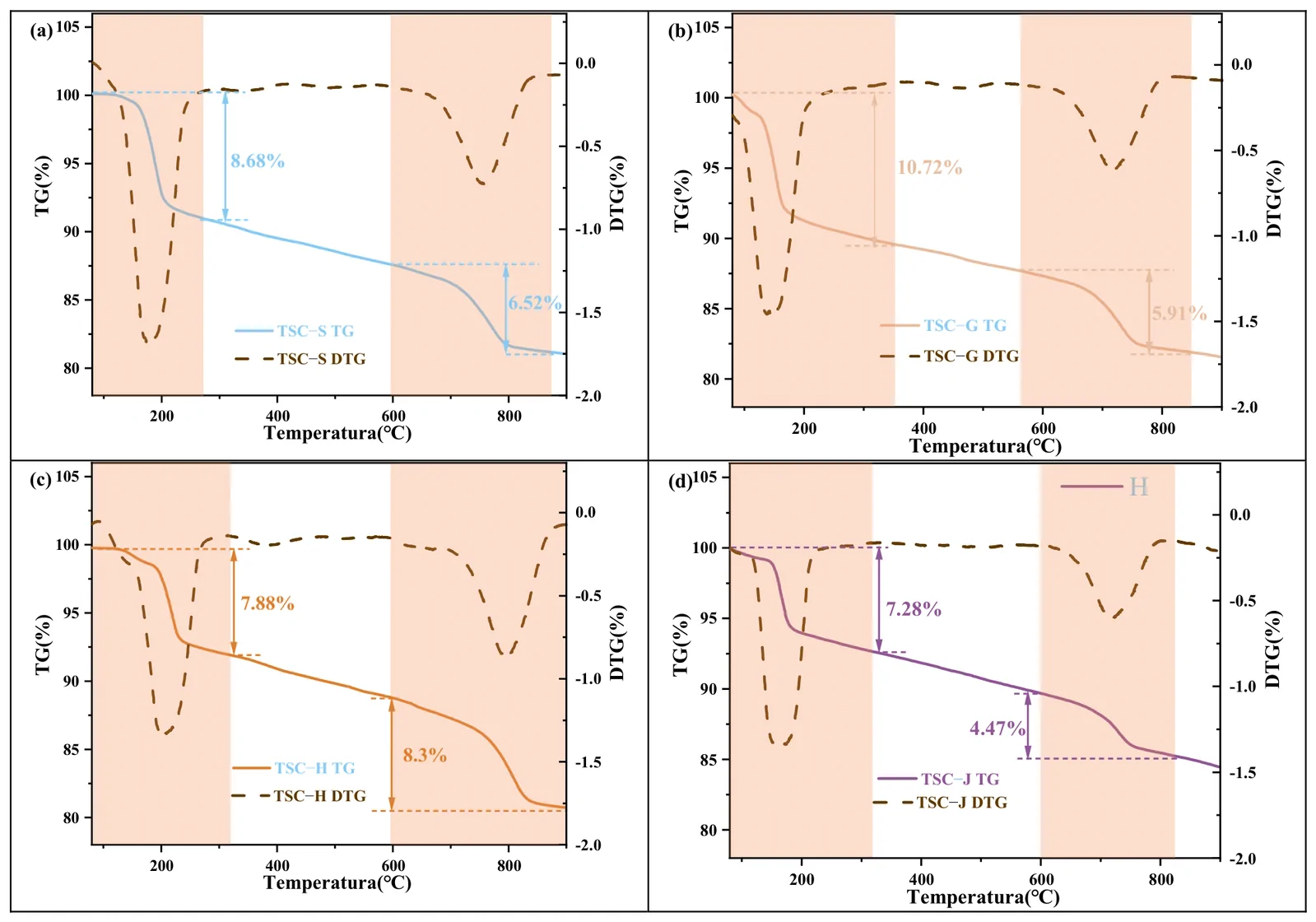
TG and DTG diagrams of different curing methods: (**a**) TG and DTG diagrams of TSC-S; (**b**) TG and DTG diagrams of TSC-G; (**c**) TG and DTG diagrams of TSC-H; (**d**) TG and DTG diagrams of TSC-J.

Figure 9a presents the TG and DTG curves of the cement-cured (TSC-S) sample. The TG diagram indicates that TSC-S exhibits an overall mass loss of approximately 8.68%, with notable reductions occurring in the 200–400 °C and 600–800 °C temperature ranges. The DTG spectrum shows that within 200–400 °C, TSC-S undergoes substantial mass loss, primarily attributed to water evaporation and dehydration of bound water in C-S-H gel. In the high-temperature range of 600–800 °C, mass loss is mainly associated with the decomposition of CaCO_3_, releasing CO_2_ and forming CaO. This decomposition appears as a distinct mass loss peak in the DTG curve [68]. Overall, the cement-cured system exhibits relatively low thermal stability, particularly due to the pyrolysis of hydration products. For the solid waste-based solidification (TSC-G) system, Figure 9b indicates an overall mass loss of 10.72%, with 5.91% occurring in the 200–400 °C range. The DTG curve reveals a relatively high mass loss rate in this temperature range, suggesting that water evaporation and mineral hydration in the solid waste-based cementitious system predominantly govern mass loss [69]. Overall, TSC-G exhibits relatively good thermal stability, which can be attributed to the formation of low-crystallinity, silica-rich aluminum gels (C-(A)-S-H and N-A-S-H). The low crystallinity of these gels slows their decomposition at elevated temperatures [70]. DTG analysis shows that mass loss of TSC-G in the 600–800 °C range is relatively low, indicating mild pyrolysis behavior. Figure 9c presents the TG and DTG curves of the lime-cured (TSC-H) sample. The TG curve indicates an overall mass loss of 7.88% for TSC-H, with 8.3% occurring in the 200–400 °C range. This mass loss is primarily associated with water evaporation during lime curing and the hydration of calcium hydroxide (Ca(OH)_2_) [71]. DTG analysis further indicates a relatively high mass loss rate in this range, reflecting the dehydration of lime hydration products. Mass loss between 600 and 800 °C corresponds to CaCO_3_ decomposition. The overall mass loss remains relatively low, indicating that the lime-cured soil exhibits good thermal stability, although lower than that of cement- and solid waste-based systems. Figure 9d presents the TG and DTG curves of microbial-cured (TSC-J) soil. The overall mass loss of TSC-J is 7.28%, with 4.47% occurring in the 200–400 °C range. The DTG spectrum shows that microbial-cured samples exhibit a relatively high mass loss rate in this range, suggesting that moisture removal and volatilization of microbial metabolic products dominate the pyrolysis processes. Microbial-cured soil demonstrates relatively high thermal stability, particularly at elevated temperatures, where the stability of CaCO_3_ reduces mass loss [72]. TG and DTG analyses indicate that the thermal stability of TSC composite soil varies significantly with the curing method. The cement-cured system (TSC-S) exhibits substantial mass loss, particularly in the 200–400 °C range, primarily due to water evaporation and dehydration of hydration products. The solid waste-based system (TSC-G) demonstrates high thermal stability, largely owing to the low-crystallinity gel structure, which decomposes slowly at elevated temperatures. The lime-cured system (TSC-H) exhibits relatively mild pyrolysis, predominantly governed by hydration and carbonation reactions. Microbial-cured soil (TSC-J) shows good thermal stability, particularly at elevated temperatures, where the stability of CaCO_3_ enhances the soil’s heat resistance. Overall, TG and DTG results highlight the influence of curing methods on the thermal stability of TSC composites, providing a fundamental basis for further investigations into the structural stability and mechanical behavior of each system.

#### 3.2.3. Microstructural Characteristics

**Figure 10:**
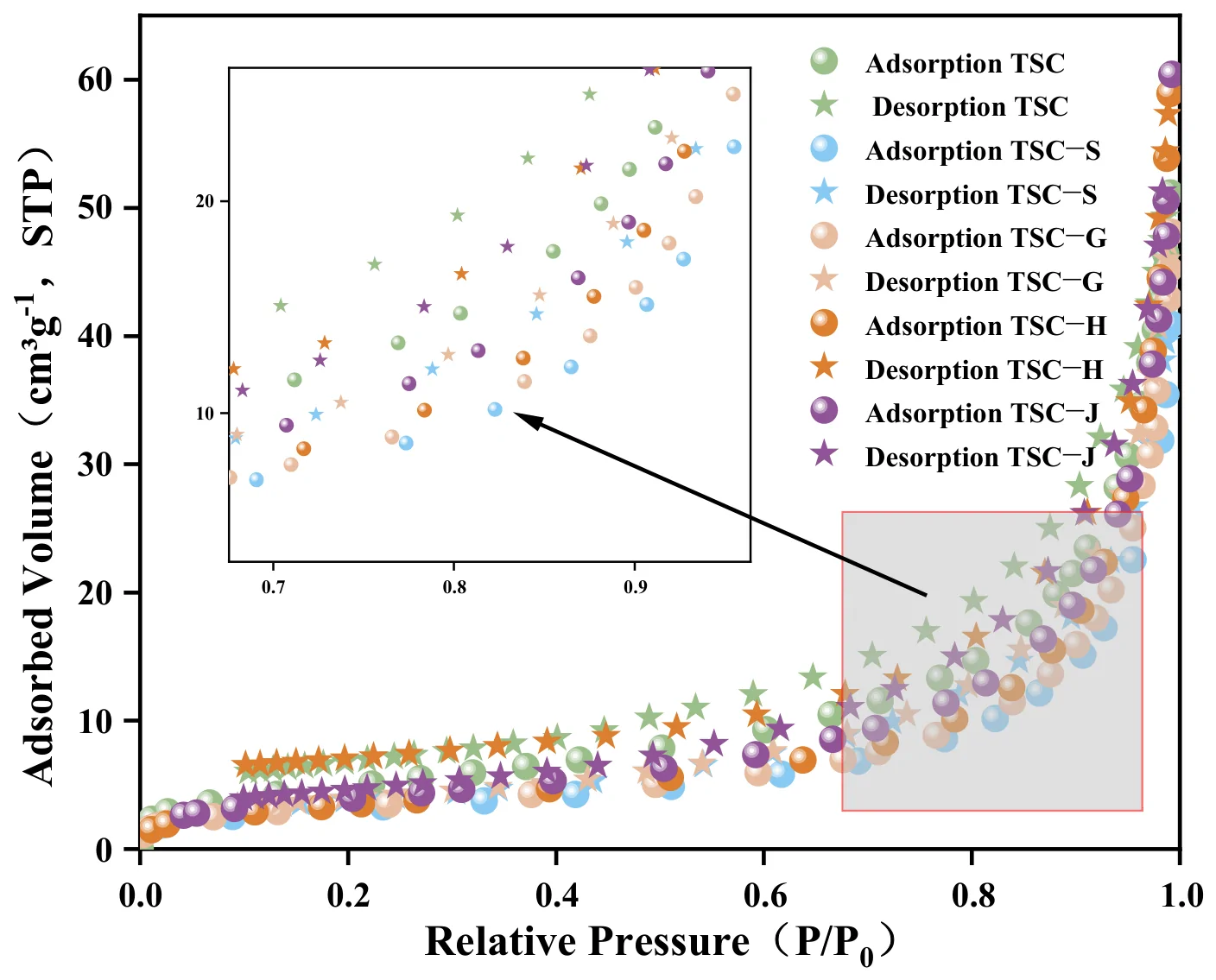
Nitrogen adsorption–desorption isotherms of different curing methods.

To investigate the effects of different curing methods on the microstructure of TSC composite soil, porosity and pore distribution were analyzed using BET and SEM techniques. Microstructural characteristics are critical for understanding soil strength, porosity, and stability. The evolution of soil structure under different curing agents significantly affects macroscopic properties. Figure 10 presents the nitrogen adsorption–desorption isotherms of TSC composite soil for different curing methods. All samples exhibit a pronounced increase in adsorption at low relative pressures (P/P_0_ < 0.1), indicating the presence of microporous structures in the cured TSC composite soil [73]. The TSC-H and TSC-J samples display a notable increase in adsorption at high relative pressures, suggesting higher specific surface areas, greater porosity, and stronger adsorption capacity. In contrast, TSC-S and TSC-G exhibit relatively smooth adsorption curves, indicating lower porosity and a denser microstructure.

**Figure 11:**
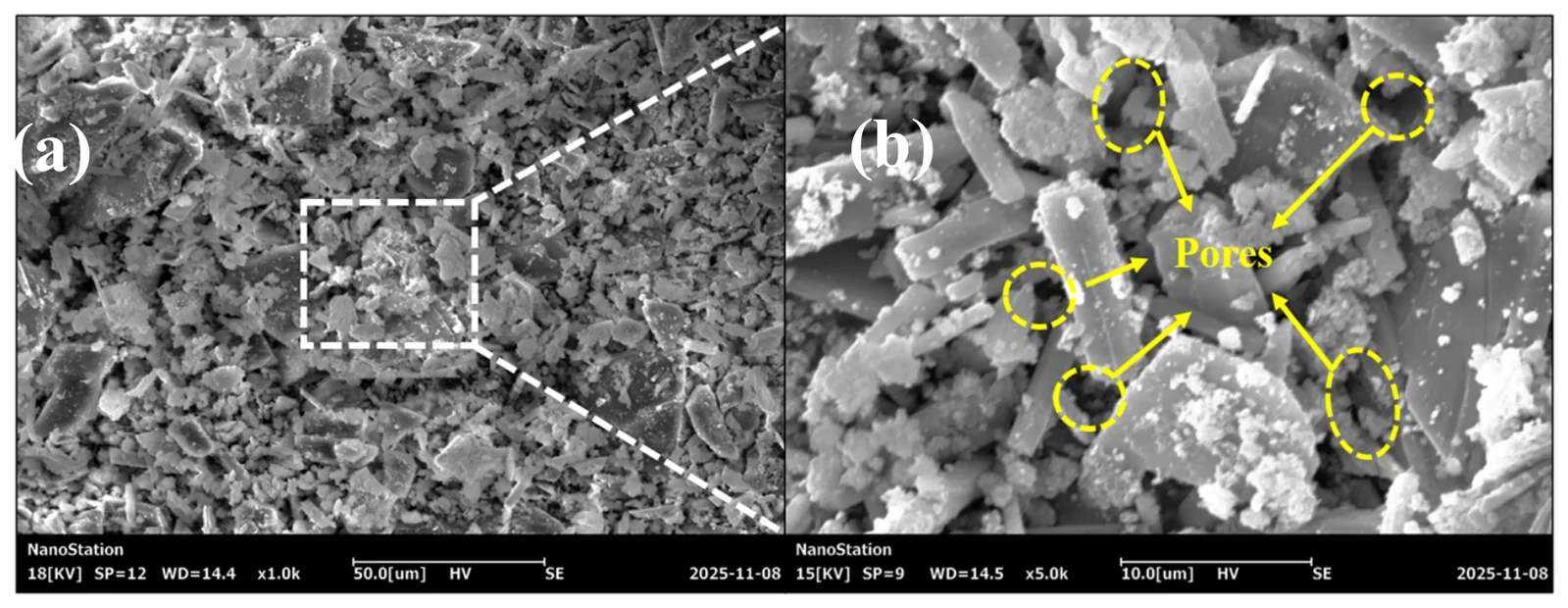
SEM image of uncured TSC composite soil: (**a**) microscopic image and (**b**) magnified local image.

Figure 11 presents the SEM image of the uncured TSC composite soil. SEM observation reveals that the uncured soil exhibits a well-defined pore structure, characterized by relatively loose particle interfaces and a fairly uniform pore distribution. The presence of these pores and irregular particle morphologies indicates that the uncured soil possesses high porosity and a relatively loose microstructure.

**Figure 12:**
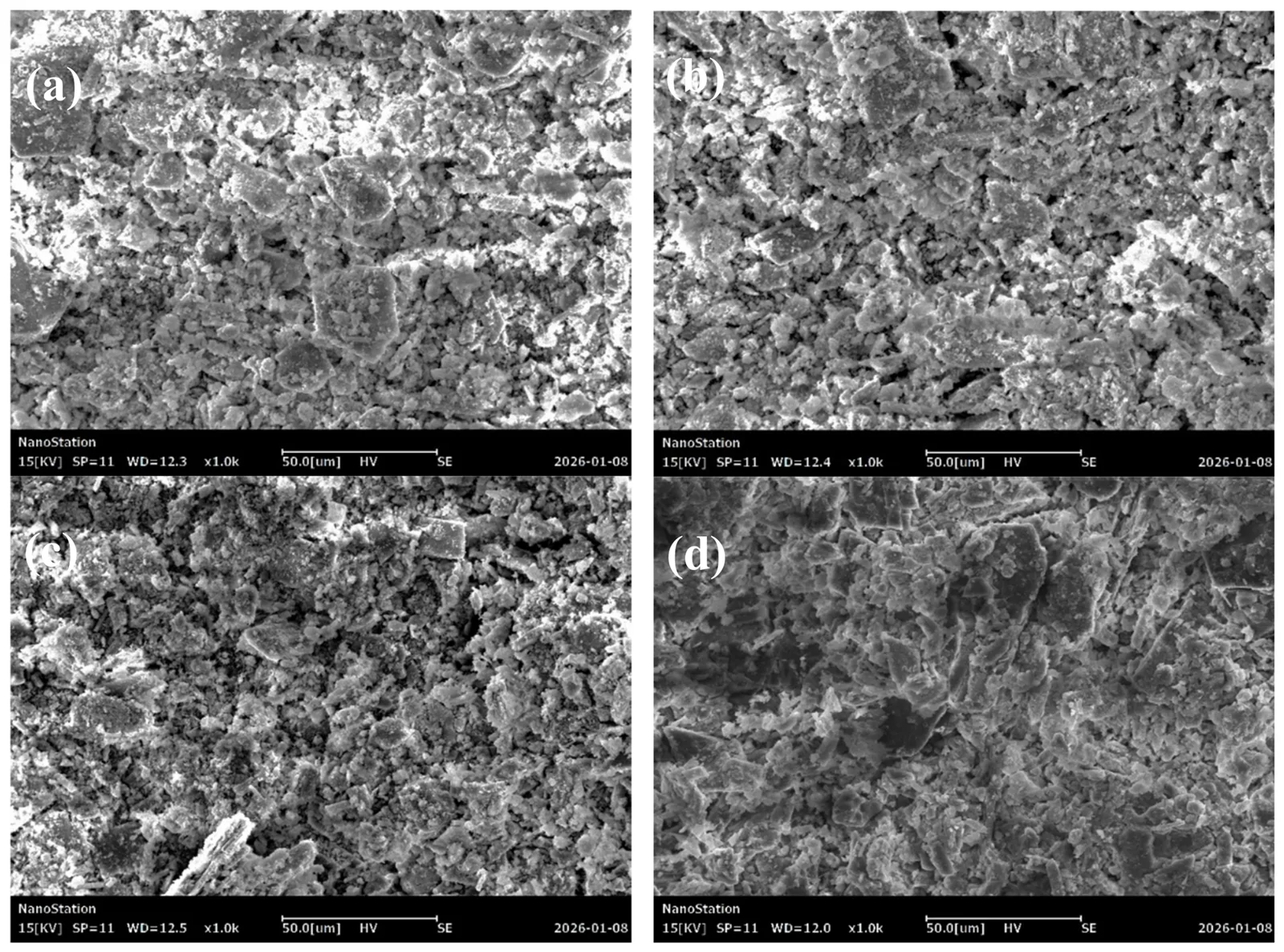
SEM images of TSC composite soil cured by different curing methods: (**a**) SEM image of TSC-S; (**b**) SEM image of TSC-G; (**c**) SEM image of TSC-H; (**d**) SEM image of TSC-J.

**Figure 13:**
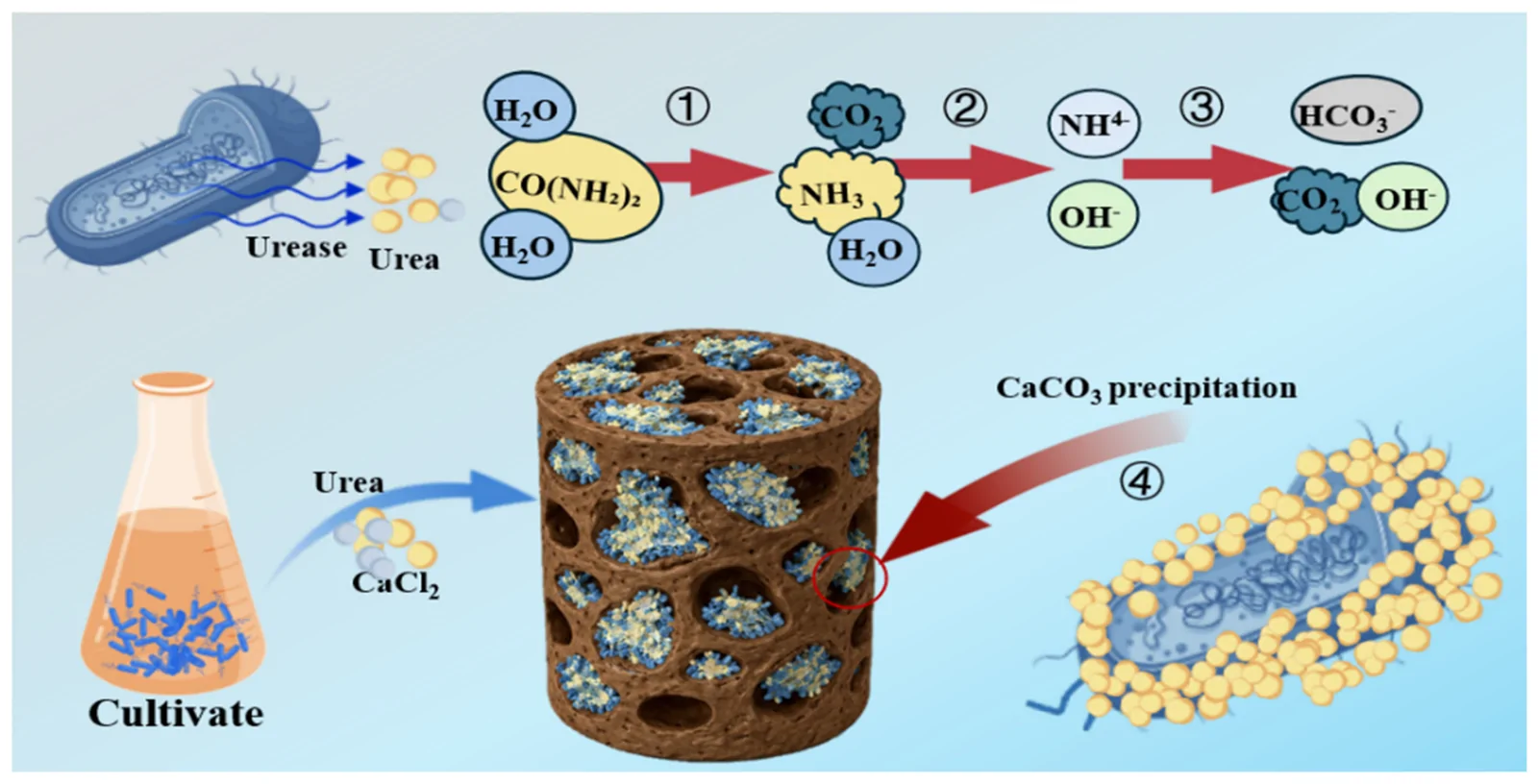
Diagram of microbial curing mechanism.

Figure 12 illustrates the SEM images of TSC composite soil after curing. Figure 12a shows that TSC-S samples exhibit a relatively dense microstructure, with tightly connected particles and pores partially filled with cementitious products, leading to a notable reduction in porosity. This indicates that cement hydration products, such as C-S-H gel, form a continuous cementing phase, effectively filling pores and enhancing interparticle bonding. These microstructural changes are consistent with the nitrogen adsorption results. In TSC-G samples (Figure 12b), particle surfaces exhibit a denser cementation network, fewer pores, and a more uniform gel distribution. This suggests that the alkali-activated reaction of solid waste-based materials generates C-(A)-S-H gels, forming a robust cementation network on particle surfaces, thereby reducing porosity and enhancing soil stability. In contrast, TSC-H samples (Figure 12c) have a relatively dispersed cementing phase, more pronounced pores, and a looser microstructure. These pores are likely associated with Ca(OH)_2_ formed during lime curing. Although lime reactions provide some cementation, the resulting products do not form a robust network comparable to cement hydration products [74], resulting in a looser microstructure and higher porosity. For TSC-J samples (Figure 12d), microorganism-induced CaCO_3_ deposits form bridging structures between particles. Although pores remain, they are effectively filled, and particle connections are relatively tight. These CaCO_3_ deposits enhance structural stability, effectively fill pores, and reduce water mobility [75]. The complete mineralization process is shown in Figure 13 and can be divided into four consecutive stages: ① Urea is hydrolyzed under the action of urease to form NH_3_ and CO_2_; ② NH_3_ combines with water to form NH_4_^+^ and OH^−^, promoting a rapid increase in the pH value of the system; ③ After the system is gradually alkalized, the CO_2_/HCO_3_^−^ equilibrium shifts towards CO_3_^2−^ and accumulates; ④ CO_3_^2−^ and Ca^2+^ preferentially nucleate and deposit on the surface of the bacteria and in the surrounding pores, eventually forming CaCO_3_ crystals.

### 3.3. Analysis of Carbon Emissions

In geotechnical engineering and soil improvement, the life-cycle carbon emissions of solidified materials serve as key indicators for evaluating their environmental performance and sustainability. This study employs the Life Cycle Assessment (LCA) [12] method to systematically compare the carbon emission characteristics and environmental impacts of lime, cement, solid waste-based, and microbial solidification technologies across the stages of raw material acquisition, manufacturing, transportation, application, and end-of-life treatment. To quantitatively evaluate carbon emissions over the entire life cycle of different curing systems, the emission factor method [76] was employed, with its fundamental formula presented in Equation (2):

(2) $$C_{\mathrm{total}}=\sum_{i=1}^{n}M_{i}\times EF_{i}$$

In the formula, *C*_total_ denotes the total carbon emissions (kg CO_2_) of the curing system over its life cycle; *M_i_* is the mass (kg) of the i-th material; *EF_i_* represents the carbon emission factor (kg CO_2_ kg^−1^) of the corresponding material; and *n* is the total number of material types included in the system.

To enable comparison among different solidification technologies, the carbon emissions are normalized as the carbon emission intensity per unit mass of soil, with the calculation formula presented in Equation (3):

(3) $$C_{\mathrm{unit}}=\frac{C_{\mathrm{total}}}{M_{\mathrm{soil}}}$$

In the formula, *C*_unit_ denotes the carbon emission per unit mass of soil (kg CO_2_·t^−1^), and *M*_soil_ represents the total mass (t) of solidified soil in the sample.

In the microbial-induced carbonate precipitation (MICP) system, microbial mineralization produces calcium carbonate and sequesters part of the carbon dioxide. Therefore, it is essential to calculate the system’s net carbon emissions, as expressed in Equation (4).

*C_net_* = *C_total_* − *C_fix_* (4)

Here, *C*_net_ denotes the system’s net carbon emissions (kg CO_2_), and *C*_fix_ denotes the amount of CO_2_ (kg CO_2_) sequestered during microbial mineralization.

The amount of carbon sequestered can be calculated from the mass of calcium carbonate produced. The corresponding formula is given in Equation (5).

(5) $$C_{\mathrm{fix}}=M_{\mathrm{CaC}O_{3}}\times \frac{44}{100}$$

Here, $M_{{\mathrm{CaCO}}_{3}}$ denotes the mass (kg) of calcium carbonate produced, and 44/100 represents the mass fraction of CO_2_ in the CaCO_3_ molecule.

Using the above calculation method, the carbon emission intensity and carbon sequestration potential of different curing systems can be systematically assessed, enabling a quantitative comparison of their environmental performance.

Cement production is associated with substantial CO_2_ emissions, primarily from limestone calcination and fuel combustion. The carbon emission intensity of conventional Portland cement is approximately 0.8–1.0 t CO_2_ per ton of cement, with a major contribution from limestone decomposition [77]. Accordingly, the carbon footprint of cement-based stabilization includes emissions from material production, energy consumption, and transportation.

**Figure 14:**
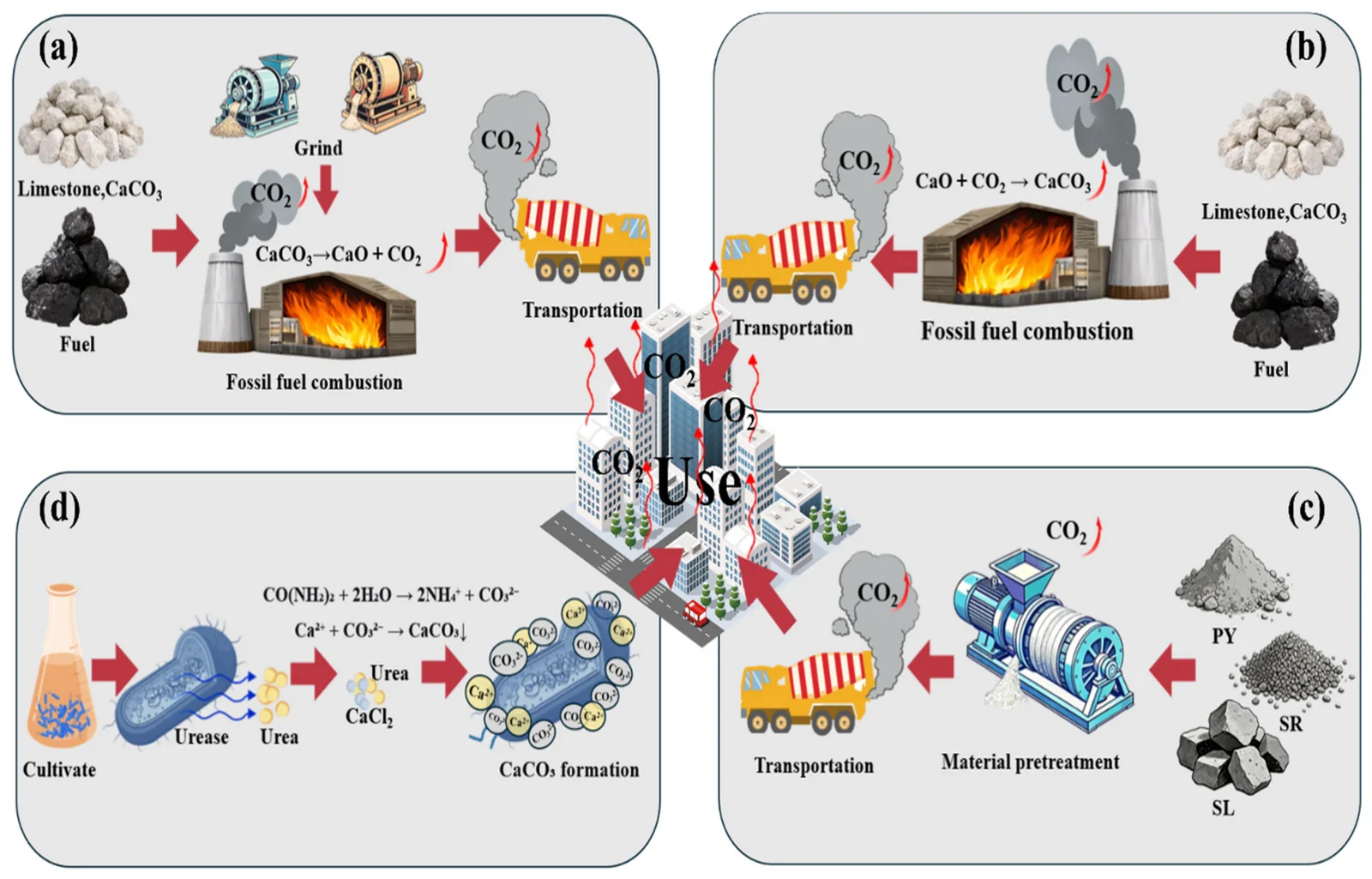
Life cycle carbon emissions of different stabilization systems.

Lime (primarily calcium oxide, CaO), a traditional soil stabilizer, exhibits life cycle emissions similar to cement, though slightly lower. Lime production similarly requires high-temperature calcination of natural limestone to produce CaO, releasing substantial CO_2_. The overall process is shown in Figure 14b. Life cycle inventory studies indicate that approximately 0.79 tons of CO_2_ are emitted per ton of calcium oxide produced, with the calcination process contributing the majority of these emissions [78]. Although lime application in soil stabilization emits less CO_2_ than pure cement systems, its manufacturing and transportation stages remain the primary contributors to life cycle carbon emissions. Therefore, the low-carbon advantages of lime curing primarily arise from enhanced curing efficiency and partial replacement of cement-based systems; however, emissions from manufacturing and energy use remain significant.

With the growing demand for industrial solid waste utilization (e.g., fly ash, granulated blast furnace slag, steel slag, desulfurization gypsum), solid waste-based cementitious materials have emerged as a key focus for low-carbon solidification technologies. By partially or fully replacing traditional cement clinker with industrial by-products, these materials reduce primary energy and raw material consumption while effectively lowering carbon emissions in associated processes. Compared with ordinary Portland cement, solid waste-based cementitious materials can reduce CO_2_ emissions by 40–80% [79], largely due to the absence of high-temperature calcination and their potential carbon sequestration effect. Life cycle carbon assessment should also consider pretreatment energy from different waste sources, transportation distances, and activator use. Overall, the environmental impact of solid waste-based materials is significantly lower than that of conventional cement-lime solidification systems. The overall process is shown in Figure 14c.

Microbially induced calcium carbonate precipitation (MICP) is a biomineralization-based soil stabilization technique in which microbial activity promotes the formation of CaCO_3_ deposits, improving interparticle bonding and soil structure. Its environmental performance depends on the consumption of raw materials and energy, microbial cultivation, and the carbon sequestration associated with carbonate precipitation. Therefore, a life-cycle perspective is necessary to evaluate its net environmental benefits. Life cycle assessment (LCA) results indicate that, although MICP consumes raw materials and energy—and in some applications its carbon emissions exceed those of traditional materials—the raw material preparation stage dominates total emissions. This underscores the need to further reduce environmental loads through careful raw material selection, optimized microbial cultivation, and process improvements. Additionally, the CaCO_3_ generated by MICP has inherent carbon sequestration potential. During biomineralization, CO_2_ is converted into stable carbonate minerals, offering carbon sequestration advantages over traditional solidification systems. When combined with renewable calcium and suitable carbon sources, MICP can achieve a substantial reduction in net carbon emissions. Therefore, evaluating MICP’s environmental performance from a full life cycle perspective requires comprehensive consideration of carbon emissions from raw material preparation, energy consumption during construction, and carbon sequestration benefits throughout the product life cycle.

To systematically evaluate environmental performance, this study developed a three-in-one assessment framework encompassing carbon emissions, carbon sequestration, and overall environmental benefits for four mainstream solidification methods: cement, lime, solid waste-based, and microbial. Using a 5% dosage as the benchmark, life cycle assessment (LCA) was applied to quantify the full-process carbon footprint, with key results presented in Table 4. Cement curing exhibits the highest carbon emissions at 45 kg CO_2_ per ton, primarily due to high energy consumption during limestone calcination and production [80]. Lime curing produces 35 kg CO_2_ per ton; although lower than cement, substantial emissions still occur during calcination and transportation [81]. Carbon emissions from solid waste-based solidification have decreased to 12.8 kg CO_2_ per ton, benefiting from the carbon sequestration potential of industrial by-products [13]. Microbial mineralization and solidification demonstrate a unique synergy between emission reduction and carbon sequestration. The urea hydrolysis process generates 13.8 kg CO_2_ per ton [82], whereas microbially induced calcium carbonate precipitation fixes 9.5 kg CO_2_ per ton [83], resulting in a net emission of only 4.3 kg CO_2_ per ton—a 66.7–87.7% reduction compared with the other technologies.

**Figure 15:**
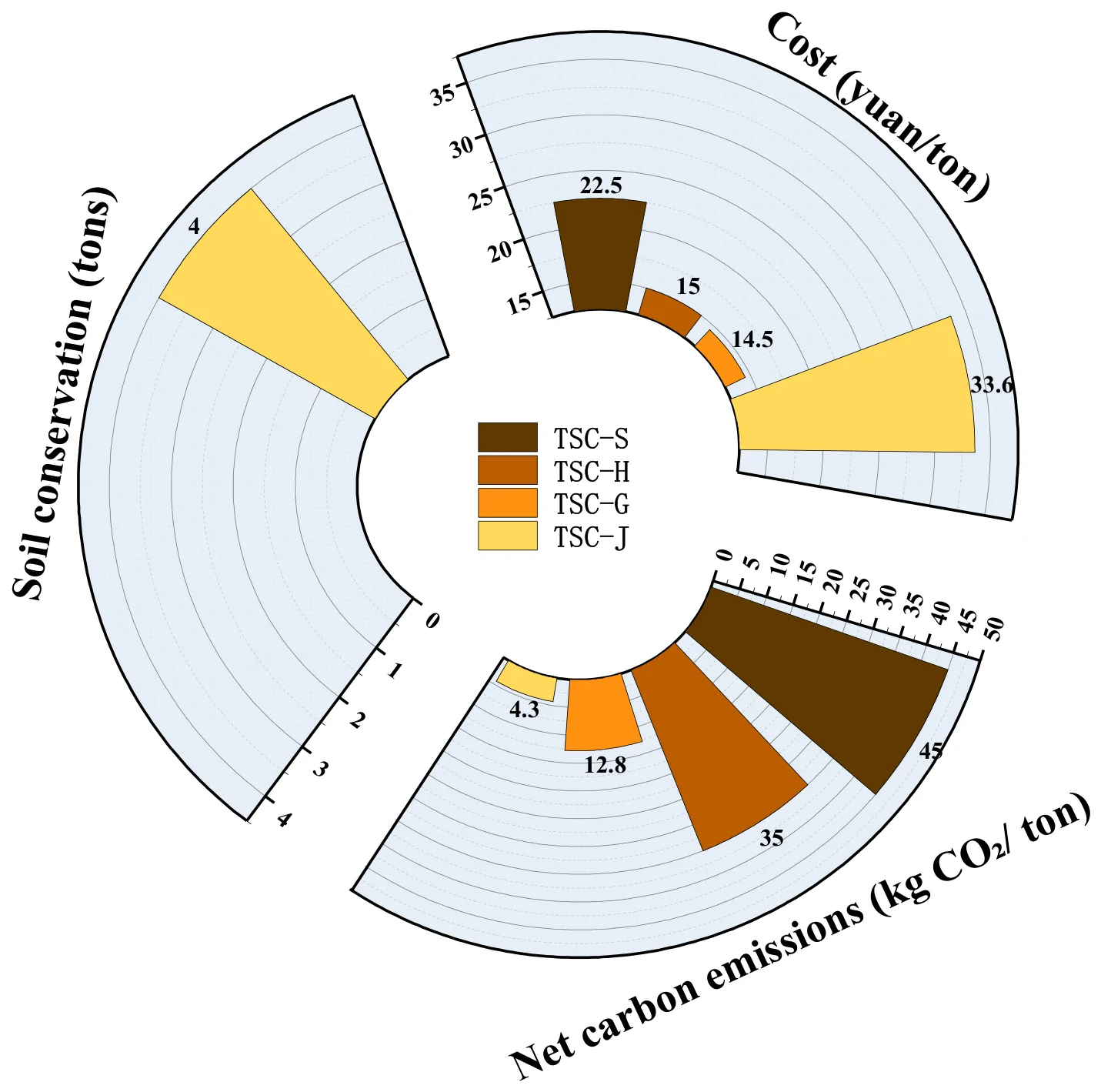
Comparison of economic and ecological benefits of four soil fixation methods.

Additionally, microbial mineralization curing demonstrates superior performance across multiple metrics: its unit energy consumption is only 180 kWh per ton [84], representing 20.7% of that for cement curing, and its mild reaction conditions eliminate the need for high-temperature calcination. The precipitated calcium carbonate can form a coating and adsorb heavy metals, reducing the leaching of lead and cadmium ions by 40–60% compared with cement curing [85]. At the resource recycling level, microbial curing enables multi-objective coordination of solid waste disposal, carbon sequestration, soil resource conservation, and performance enhancement. Moreover, replacing 30–50% of conventional clay fillers with titanium gypsum allows the annual consumption of 50,000 tons of titanium gypsum to reduce clay mining by 25,000–30,000 tons, equivalent to 33,000–40,000 m^3^ of high-quality soil resources, effectively reducing the dependence of roadbed construction on natural soil resources and mitigating soil erosion and ecological damage [86]. Calculations show that the clay–MICP composite modification technology developed in this study (Figure 15) costs no more than 80 yuan per ton of modified soil, 17.2% lower than conventional cement solidification. At the assumed implementation scale, the proposed approach could potentially increase titanium-gypsum utilization and reduce the demand for natural soil resources. However, these potential resource and economic benefits require validation under actual engineering and regional operating conditions. Furthermore, the net carbon emission per ton of solidified soil is only 4.3 kg CO_2_, 86% lower than cement solidification. With global adoption, the annual carbon reduction potential exceeds 500,000 tons, providing critical support for titanium dioxide enterprises in achieving the dual objectives of zero solid waste storage and carbon reduction.

Life cycle assessment (LCA) of four soil solidification technologies indicates that traditional cement solidification is mature and provides relatively high strength, but exhibits the highest carbon footprint, primarily due to limestone calcination and fuel consumption. Carbon emissions from lime solidification are slightly lower than those of cement, but remain dominated by high-temperature calcination, offering limited low-carbon advantages. Solid waste-based solidification materials significantly reduce carbon emissions and represent an important current research focus for low-carbon solidification. Microbial solidification (MICP) leverages biomineralization and carbon sequestration mechanisms. Although emissions are influenced by raw materials and process parameters, MICP’s carbon sequestration potential and environmental benefits are prominent, positioning it as a key direction for future low-carbon solidification technologies. The present results suggest that MICP has potential as a lower-carbon stabilization approach for TSC composite soil under the assessment conditions adopted in this study. However, its environmental and engineering advantages remain dependent on reagent production, microbial cultivation, transportation, energy consumption, treatment uniformity, and long-term field performance. Therefore, its application to subgrade, slope, and other geotechnical engineering scenarios should be further evaluated through field-scale trials and comprehensive techno-economic and life-cycle assessments.

## 4. Conclusions

This study systematically compared the laboratory-scale performance of cement, lime, solid-waste-based cementitious materials, and microbial-induced calcium carbonate precipitation (MICP) for the stabilization of TSC composite soil. The main conclusions are summarized as follows:

(1) Cement-solidified TSC composite soil exhibited the highest mechanical performance at 28 days, with an unconfined compressive strength (UCS) of 2.6 MPa, a water stability coefficient of 0.88, and a post-freeze–thaw strength of 2.02 MPa, indicating the best durability. However, cement stabilization showed the highest estimated carbon emissions, at 45 kg CO_2_/t under the adopted assessment assumptions.

(2) Solid waste-based cementitious materials achieved a 28-day UCS of 1.94 MPa and a water stability coefficient of 0.82, demonstrating good overall mechanical performance and offering the advantage of solid waste utilization. The estimated carbon emissions were approximately 12.8 kg CO_2_/t under the adopted assessment assumptions, substantially lower than those of the cement-stabilized system.

(3) Lime-curing resulted in a 28-day UCS of 1.57 MPa and a water stability coefficient of 0.77, indicating weaker mechanical properties and lower freeze–thaw durability. The estimated carbon emissions were 35 kg CO_2_/t under the adopted assessment assumptions, representing only a moderate reduction relative to cement stabilization.

(4) Microbial solidification (MICP) achieved a 28-day UCS of 1.12 MPa and a water stability coefficient of 0.79. Although its short-term mechanical performance was lower than that of the other stabilization systems, CaCO_3_ precipitation contributed to pore filling and interparticle bonding. Under the adopted life-cycle assessment assumptions, the estimated net carbon emission was 4.3 kg CO_2_/t, approximately 86% lower than that of cement stabilization. These results indicate the potential of MICP as a lower-carbon stabilization approach, although its engineering feasibility requires further field-scale validation.

Overall, the results demonstrate clear trade-offs among mechanical performance, durability, and estimated carbon emissions for the four stabilization approaches. Cement and solid-waste-based binders provided relatively superior mechanical performance under the tested conditions, whereas MICP showed lower short-term strength but a lower estimated net carbon emission. These findings provide a comparative basis for selecting stabilization strategies for TSC composite soil, rather than establishing the direct superiority or field-scale applicability of any single method.

The present study is subject to several limitations. First, all experiments were conducted under controlled laboratory conditions, with a maximum curing age of 28 days, and durability was evaluated using a limited number of freeze–thaw cycles. Therefore, the long-term performance of stabilized TSC soil under actual field conditions remains uncertain. Second, the estimated carbon emissions depend on the adopted system boundaries, emission factors, transportation and energy assumptions, and the estimation of carbon sequestration associated with CaCO_3_ formation. In addition, raw-material variability, MICP treatment uniformity, construction-scale feasibility, and comprehensive economic performance were not fully evaluated. Future studies should therefore include longer-term field trials, expanded durability testing under multiple environmental exposures, optimization of microbial treatment and reagent use, and more comprehensive life-cycle and techno-economic assessments before large-scale engineering application.

## Figures and Tables

**Table 1:** Chemical composition of the raw materials (wt.%).

Material	SO_3_	CaO	Fe_2_O_3_	SiO_2_	TiO_2_	Al_2_O_3_	Others
Titanium gypsum	34.4	32.3	6.3	4.9	3.7	2.5	15.9
Clay	-	-	1.14	64.47	-	23.38	11.01

**Table 2:** Basic physical properties of titanium gypsum and TSC composite soil.

Material	Liquid Limit W_L_ (%)	Plastic Limit W_P_ (%)	Dry Density (kg·m^−3^)	Free Swelling Rate (%)
TS	67.82	30.96	1128.79	42
TSC	46.71	23.91	1427.03	29.5

**Table 3:** Experimental groups of different curing agents.

Test Number	TSC-S	TSC-G	TSC-H	TSC-J
Curing Agent	cement	solid waste base	lime	microorganism
Dosage (%)	5%	5%	5%	40% dosage

**Table 4:** Comparison of carbon emissions from four curing methods.

Curing Method	Material Cost (CNY/t)	Carbon Emissions (kg CO_2_/t)	Carbon Sequestration (kg CO_2_/t)
TSC-S	22.5	45.0	0
TSC-H	15.0	35.0	0
TSC-G	14.5	12.8	0
TSC-J	33.6	13.8	9.5

## Data Availability

The original contributions presented in this study are included in the article. Further inquiries can be directed to the corresponding author.
